# Using sodium glycodeoxycholate to develop a temporary infant-like gut barrier model, *in vitro*

**DOI:** 10.3389/fnut.2025.1577369

**Published:** 2025-06-09

**Authors:** Francesca Bietto, Elena Arranz, Beatriz Miralles, Cristina Gómez-Marín, Eva Rath, Alice J. Lucey, Linda Giblin

**Affiliations:** ^1^Teagasc Food Research Centre, Moorepark, Cork, Ireland; ^2^School of Food and Nutritional Sciences, University College Cork, Cork, Ireland; ^3^Sección Departamental de Ciencias de la Alimentación, Universidad Autónoma de Madrid (UAM), Madrid, Spain; ^4^Institute of Food Science Research CIAL (CSIC-UAM), Madrid, Spain; ^5^Chair of Nutrition and Immunology, School of Life Sciences, Technische Universität München, Freising, Germany

**Keywords:** *in vitro* infant gut barrier, sodium glycodeoxycholate, Caco-2/HT29-MTX, tight junctions, intestinal permeability, TEER, infant milk formula digesta

## Abstract

**Introduction:**

In newborns, the intestinal barrier is permeable but not inflamed. Understanding this unique state is essential for developing models relevant to infant gut physiology.

**Methods:**

This study aimed to develop an *in vitro* model of the infant gut barrier treating Caco-2/HT29-MTX with 0.5, 0.8, and 1 mM sodium glycodeoxycholate (GDC).

**Results:**

Our research demonstrates that GDC decreases Caco-2/HT29-MTX Trans-Epithelial Electrical Resistance (TEER) and increases paracellular permeability, without inflammation or cytotoxicity. Notably, the treatment with 0.8 mM GDC increased lactulose transport rate by 1.63-fold. The treatment also reduced the key tight junction protein, occludin, at the cell membrane, and increased acidic mucins and extracellular alkaline phosphatase activity. Additionally, GDC decreased cAMP, suggesting its mechanism of action was via activation of a G-protein coupled receptor. Of particular importance to nutrition studies, the GDC effect was reversible with TEER recovery within 4 h. Applying digested infant formula to 0.8 mM GDC-treated Caco-2/HT29-MTX monolayers resulted in a higher concentration of amino acids in the basolateral compartment compared to control monolayers.

**Discussion:**

These findings suggest that GDC can modulate gut barrier properties in a controled, reversible manner, offering a valuable model for studying nutrient absorption and gut physiology in early life.

## Graphical Abstract

Created with BioRender.com. 
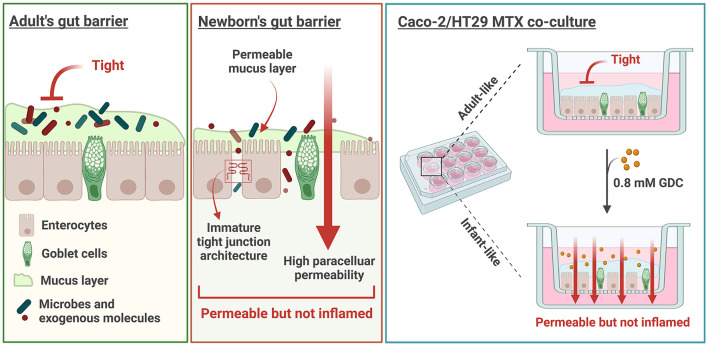

## Highlights

- GDC temporarily decreases TEER in Caco-2/HT29-MTX monolayers, without cytotoxicity
- GDC decreases OCLN fluorescence intensity and increases lactulose paracellular transport
- GDC increases acidic mucins production and decreases intracellular cAMP
- GDC treated monolayers are biocompatible with Infant Formula digesta

## 1 Introduction

The infant gut barrier has a higher paracellular permeability during early development (1–3), facilitating the absorption of bioactive molecules from human milk (4). Human milk, in turn, matures the gut barrier and gradually reduces its permeability overtime (1). Infant formulas are formulated to provide nutrients similarly to breast milk. However, the ability of breast milk to support the transition from an infant gut barrier to an adult barrier, occurs due to its unique composition of bioactive components [ie peptides (5), lipids (6, 7) oligosaccharides (8), probiotics and prebiotics].

There are ethical and logistical challenges of conducting clinical trials in early infancy to understand the gut barrier. As an alternative, current models for studying the infant gut barrier include cell lines, enteroids and animal models. Adult-derived colorectal cell lines (Caco-2, HT29-MTX) are widely used (9, 10) but differ significantly from neonatal physiology due to differences in paracellular pore size (11). The neonatal piglet-derived IPEC-J2 cell line is closer to infant gut characteristics yet limited by species-specific differences (12). Human enteroids closely mimic infant intestinal physiology but face challenges related to culture complexity and scalability (13). Animal models (neonatal rodents, piglets) offer valuable physiological insights but ethical concerns and interspecies variations limit direct human applicability (14). These limitations underscore the necessity for developing specialized, reproducible, and infant-specific *in vitro* models of the gut barrier. Furthermore, for nutrition research, any infant-like model should be able to transition from infant to adult within a suitable timeframe, and should be able to generate data on nutrient absorption for both life stages.

The infant intestinal paracellular permeability is measured *in vivo* by quantifying lactulose (radius = 9.5 Å) and mannitol (radius = 6.7 Å) in urine after oral administration (1–3). This test represents the only direct method in humans to evaluate intestinal permeability. In healthy adults, the jejunum restricts the passage of molecules > 8 Å (15), making lactulose transport an indicator of gut barrier impairment (16). In newborns, lactulose permeability is 55.56% higher than in older infants (3 vs. 15 months = 0.28% vs. 0.18%) (2). Moreover, the lactulose-to-mannitol ratio inversely correlates with age (3, 16, 17), indicating that larger molecules, such as lactulose, can more readily cross the intestinal barrier during infancy compared to older children.

Due to the limited research in children, much of our understanding of the infant gut barrier comes from animal models. Studies using mice, for example, show that newborns can absorb big molecules, such as FITC-dextran 70 kDa (4 fold increase in transport at 2 week-old vs. 12 week-old mice) and lactoferrin 80 kDa, (4-fold increase in transport at 2 week-old vs. 12 week-old mice) (18).

Paracellular transport across the epithelial barrier is regulated by tight junction proteins, which form cell-to-cell complexes and depend on calcium for assembly (19). Key proteins maintaining barrier integrity and limiting paracellular permeability include Occludin (OCLN), Zonula Occludens (ZO-1), several Claudins (CLDN) and Junctional Adhesion Molecules (JAMs). Notably CLDN-2 increases paracellular permeability (19). In mice, mRNA expression of *Cldn3, Ocln, Tjp1* (ZO-1), and *Jam1* in the small intestine is lower in newborns (1 day and 2-week-old mice), while the expression of *Cldn2* is higher compared to adults (12-week-old mice) (18, 20).

Intestinal alkaline phosphates is a brush border enzyme expressed and secreted by enterocytes (21), whose activity is essential for maintaining gut homeostasis and protecting against inflammation (21) and aging (22). Importantly, this enzyme upregulates tight junction proteins both *in vit*ro (23) and *in vivo* (24). Interestingly, *in vivo* studies have shown that serum alkaline phosphatase levels are higher in young children (29 days to 1 year, 50^th^ percentile: males = 218.19, females = 210.62 U/L) compared to older children (17 to 18 years, 50^th^ percentile: males = 104.74, females = 75.50 U/L) (25), further supporting the enzyme's relevance during early life when the gut barrier is still developing.

Permeability is also influenced by the mucus layer, composed of high-molecular-weight glycoproteins secreted by goblet cells (26). Studies on mucus development are limited, especially in humans, but *in vivo* data show that at birth colonic mucus in humans, pigs and mice predominantly contains acidic mucins over neutral mucins (27). In human, Filipe et al. reported that acid mucins predominate in the human colon throughout fetal life. While this study does not quantify the acidic-to-neutral mucin ratio, it does suggest a predominance of acidic mucins in the neonatal period (28). In pigs, infant mucus (2-week-old) is less viscous and more permeable compared to adults (7–10-month-old) (29).

Differences in paracellular permeability, tight junctions, and mucus properties between infants and adults question the reliability of adult gut models for infant research. Given the higher incidence of necrotizing enterocolitis in formula-fed vs. breastfed infants (30, 31), developing an accurate infant gut model is crucial to understanding human milk's benefits to the young gut, and improving formulations of infant formula to mimic these benefits.

The Caco-2 monolayer (32) is a standard *in vitro* intestinal model, but it lacks a mucus layer and its tight junctions resemble those of the colon, with narrower pores (4.5 Å) (33, 34) compared to the small intestine (8–9 Å) (15). To address these limitations, the Caco-2/HT29-MTX co-culture was developed, combining enterocytes (Caco-2) and goblet cells (HT29-MTX) (10, 35) in a polarized monolayer. The 90:10 seeding ratio simulates small intestine conditions, increasing paracellular permeability and providing a mucus layer (36).

Previous studies have examined methods for increasing the permeability of Caco-2 monolayers whilst preventing inflammation. Our laboratory demonstrated that treating 20-day-old Caco-2 monolayers with 125 mM sodium butyrate effectively increases paracellular permeability without causing inflammation or cytotoxicity (37). However, the high concentration of sodium butyrate resulted in an osmolality of 0.79 Osmol/kg (37), which is at the upper limit of Caco-2 tolerance (38), possibly initiating stress responses.

For oral drug delivery studies, medium-chain fatty acids, bile salts, bacterial toxins, surfactants and chelating agents have been investigated for their ability to enhance barrier permeability (39–41). Among these molecules, sodium N-(8-[2-hydroxybenzoyl]amino) caprylate (SNAC; a C8 derivate) and sodium caprate (C10) have been widely tested in Caco-2 monolayers and clinical trials (39).

In Caco-2 (20 to 28 days old monolayers), SNAC primarily modulates the transcellular pathway, enhancing membrane fluidity but not targeting specifically tight junctions (42), which limits its relevance for modeling the paracellular permeability characteristic of the neonatal gut. On the other hand, C10 affects the paracellular permeability but requires calcium-free buffers, as it precipitates with calcium, an essential ion for tight junction maintenance and physiological relevance (34, 42, 43). Moreover, C10 acts by increasing the number of paracellular pores rather than expanding pore diameter, making it less suitable for mimicking the permeability state of the infant gut barrier (34).

Bile salts such as cholate, taurocholate, chenodeoxycholate, usodexycholate and glycodeoxucholate have been used to enhance the permeability of hydrophilic markers and drugs *in vitro* (44–47), *ex-vivo* (44, 48) and *in vivo* (45). Among these, glycodeoxycholic acid (GDC) stands out due to its favorable low toxicity and dose-dependent permeability enhancement. GDC is a secondary bile salt conjugated with glycine, synthesized through the synergistic action of the liver and gut microbiota (49, 50). In the human intestine, bile salts exist in conjugated (bound to amino acids, such as glycine or taurine) and unconjugated forms. Conjugated bile salts are more water-soluble and generally less toxic (51), whereas unconjugated forms, such as sodium cholate, tend to be more potent but are more toxic (46). GDC has been found to effectively increases permeability *in vitro* in Caco-2 monolayers (44, 52), *ex-vivo* (53) and *in vivo* (54). Specifically, 2.12 mM GDC increased calcitonin permeability in Caco-2 monolayers by 10.66-fold and decreased TEER by 4.57-fold (52), whilst 0.5 mM GDC increased [^14^C]-mannitol apparent permeability (Papp) by 3-fold and transiently decreased TEER by 2.5-fold, without reducing cell viability (MTS, CellTox™ Green assay, Caspase 3/7) (44).

Although the precise mechanism by which GDC modulates epithelial permeability is unknown, bile salts bind to TGR5 and FXR membrane receptors (55). TGR5 activation can modulate intracellular cAMP levels, which in turn regulate PKA activity (56). Deoxycholic acid, for example, signals through TGR5, leading to PKA inhibition and has been linked to EGFR activation, ERK1/2 signaling, and occludin redistribution in Caco-2 cells (57, 58). Compared to permeability enhancers like sodium butyrate (which requires high, hyperosmolar concentrations) or sodium caprate (active only in calcium-free conditions), GDC is effective at low, physiologically relevant doses (0.5–2.12 mM) under standard conditions. These properties make GDC a novel and ideal candidate for transiently inducing a leaky, infant-like epithelial phenotype, a previously unexplored approach for modeling early-life gut barrier function *in vitro*.

An *in vitro* infant model should deliver a barrier with paracellular permeability levels, tight junction and mucus characteristics similar to infants but with the capability to transition overtime to an adult barrier. Importantly, the ability of the model to recover barrier integrity after transient permeability, is essential to provide a prototype for studying gut maturation overtime, particularly when assessing dietary (bioactive) components for their ability to accelerate this transition. In this study, GDC was evaluated as a candidate for developing an infant gut barrier using Caco-2/HT-29 MTX monolayers. We hypothesized that GDC treatment would produce an infant-like barrier capable of transitioning to an adult state within a reasonably time frame for cell culture experimentation. Therefore, GDC treated monolayers could be employed in nutrition research to develop next generation infant formula.

## 2 Materials and methods

### 2.1 Materials

Human intestinal epithelial cell lines Caco-2 (EACC 86010202) and HT29-MTX-E12 (EACC 12040401) were purchased from the European Collection of Cell Culture (UK). Six and 12-wells plates and polyester permeable-membrane inserts (0.4 μm pore size, 4.5 and 1.11 cm^2^ growth area respectively) were purchased from Sarstedt Group (Germany). All pipetting was performed using low-retention, Biosphere^®^ Plus filtered pipette tips (Sarstedt, 70.3050.255). CellTiter 96™ AQueous One Solution Cell Proliferation Assay (MTS, G3582) and cAMP-Glo™ Assay kit (V1501) were purchased from Promega (USA). CyQUANT™ LDH Cytotoxicity Assay (C20300) was purchased from Invitrogen (USA). Milliplex Map Kit (HSTCMAG-28SK, Human High Sensitivity T cell Magnetic Bead Panel, was purchased from Merck Millipore (USA). RNeasy mini kit and RNA-free DNase set were from Qiagen (Netherlands). SensiFAST cDNA Synthesis Kit was from Meridian Bioscience (UK). Light Cycler SYBR GREEN I Master kit was from Roche Diagnostics (Switzerland). Primers for RT-qPCR were synthesized by Eurofins Genomics (Germany). Pierce BCA Protein Assay Kit, Rabbit Occludin Polyclonal Antibody (71–1,500), Alexafluor 488 Donkey Anti-Rabbit Antibody (A-21206), and DAPI (62,248) were purchased from ThermoFisher Scientific (USA). Infant Milk Formula (IMF) was purchased in a local supermarket. Rabbit gastric extract 15 (RGE 15) was purchased from Lipolyltech (lot number 1201, France). AccQ-Tag Ultra Derivatization Kit, Amino Acid Hydrolysate Standard, AccQ•Tag Ultra Eluent A and AccQ•Tag Ultra Eluent B were purchased from Waters Corporation^®^ (USA). All the other reagents were purchased from Sigma-Aldrich unless stated otherwise.

### 2.2 Cell culture

Caco-2 and HT29-MTX cell lines were cultured in 75-cm^2^ tissue culture flasks at 37°C, 5% CO_2_, and humidified atmosphere. After thawing, they were passaged three times before use. The culture medium was Dulbecco's Modified Eagle Medium (DMEM, D5796), supplemented with 10% fetal bovine serum (FBS, F7524), and 100 U/mL Penicillin-Streptomycin (P4333), referred to as “complete DMEM.” For HT29-MTX cells, 1% MEM Non-essential Amino Acid Solution (M7145,) was also added. Cells were passaged at 80% confluence every 4–5 days using 0.25% Trypsin-EDTA (T4049). Cells were used between passage numbers 22–28 for Caco-2, and 64–68 for HT29-MTX cells. Cells Trypan blue staining and a TC20 cell counter (Bio-Rad, USA) were used for cell counting.

### 2.3 MTS assay

GDC was dissolved in DMEM D1145 and filter sterilized using 0.22 μm PET syringe filters, (A16532-GUK, Misart Engineering Ltd, New Zealand), to a concentration of 0.5 to 5 mM. Gastrointestinal digested samples of infant milk formula (IMF) were thawed at room temperature, then diluted to ratios of 1 in 10, 1 in 15, and 1 in 20 in HBSS, followed by filtration using a 0.45 nm PES syringe filter. Caco-2 and HT29-MTX cells were seeded in a 96-well plate at a concentration of 10^5^ cells/well at a 90:10 ratio (Caco-2:HT29-MTX) in complete DMEM+1% MEM. After ~16 h the cells were washed with HBSS and incubated for 30 min with DMEM free from glutamine and phenol red (DMEM D1145). Cells were then washed twice with Hanks Balanced Salt Solution (HBSS, H8264) and incubated with 200 μL GDC (12 concentrations, 0–5 mM) in DMEM D1145 or 1% TritonX (X-100). After 2 h at 37°C, 20 μL MTS reagent were added to each well and incubated for 1 h. For the digested samples, 200 μL of pre-warmed (37°C) digesta was added for 2 h and then removed, as its turbidity interfered with absorbance readings. Cells were washed twice with HBSS and then 200 μL of MTS reagent diluted 1 in 11 in HBSS (for each reaction: 18 μL of MTS reagent + 182 μL of HBSS) was added for 1 h. Absorbance was read at 490 nm in a microplate reader (Synergy HT BioTek, Winooski, VT, USA). Cell viability was determined by normalizing the absorbance values to DMEM-treated cells (set as 100%) for GDC samples and HBSS-treated cells for digesta samples. The IC_50_ values for GDC were determined using non-linear regression analysis.

### 2.4 Caspase-3 assay

Caco-2/HT29-MTX (90:10) were seeded 10^5^ cells/well in a 96-well plate, cultured overnight and then treated with 0.5, 0.8 and 1 mM GDC for 2 h. Caspase-3 assay was performed according to the manufacturer's guidelines. Briefly, the plate was cooled on ice, media removed, wells rinsed with HBSS. Then, 200 μl reaction mixture (including peptide substrate acetyl-Asp-Glu-Val-Asp-7-amido-4-methylcoumarin) was added to the wells, mixed by pipetting and transferred into a black, flat bottom 96-well plate. Fluorescence was read (excitation—360 nm, emission—460 nm) every min for 60-min at 37°C using a plate reader (Synergy HT, Biotek). Control cells were exposed to DMEM D1145 only, and the assay positive control was isolated human 0.5 μM Caspase-3 (no cells). Caspase-3 activity was quantified in nanomoles of 7-Amino-4-methylcoumarin (the fluorescent product) liberated from acetyl-Asp-Glu-Val-Asp-7-amido-4-methylcoumarin per minute per milliliter of cell lysate, calculated using the formula:


$$\begin{array}{l} \text{Activity},\text{ nmol AMC}/\text{min}/\text{mL}=\left(\text{nmol AMC}\times \text{d}\right)/\left(\text{t}\times \text{v}\right) \end{array}$$

Where *v* = volume of the sample in milliliters, *d* = dilution factor, and *t* = reaction time in min.

### 2.5 Intracellular cAMP quantification

Cyclic AMP levels were assessed using the cAMP-Glo™ Assay (V1501, Promega), according to the manufacturer's instructions. Caco-2/ HT29-MTX (90:10) were seeded in a 96-well plate at 10^5^ cells/well and cultured overnight. On the experiment day, 100 mM IBMX (3-isobutyl-1-methylxanthine) was prepared by dissolving 22.2 mg in 100% DMSO and diluted in DMEM D1145 to a final concentration of 500 μM. Cells were washed twice with HBSS and incubated with DMEM D1145 + IBMX for 30 min to equilibrate.

During equilibration, 0.5, 0.8, and 1 mM GDC solutions were prepared in DMEM D1145 + IBMX and filter sterilized. DMEM D1145 + IBMX was removed from each well and replaced with 100 μL of the GDC in DMEM D1145 + IBMX. Cells were incubated at 37°C for 2 h. Following the incubation, 80 μL was removed from each well. Cyclic AMP-Glo™ Lysis Buffer (20 μL) was added to the remaining 20 μL volume in the wells. The plate was incubated at room temperature on a shaker for 15 min. Subsequently, a cAMP Detection Solution was prepared by mixing 2.5 μL of Protein Kinase A (PKA) with 1 mL cAMP-Glo™ Reaction Buffer. Next, cAMP Detection Solution (40 μL) was added to each well, and the plate was shaken for 30 s before incubating for 20 min at room temperature. After incubation, 80 μL room-temperature Kinase-Glo^®^ Reagent was added to each well. The plate was shaken for 30 s and incubated for 10 min. Finally, 150 μL from each well was transferred to a white flat-bottom plate, and luminescence was measured using a plate reader (Synergy HT, Biotek) with the following settings: excitation = plug, emission = hole, and gain = 135.

To determine if GDC directly inhibits PKA, GDC solutions were incubated directly with cAMP in the absence of cells. Briefly, 1 μL cAMP (1 mM) was diluted in 249 μL DMEM D1145 + IBMX (final cAMP concentration 4 μM). In a 96-well plate, 10 μL of this cAMP solution was mixed with 10 μL of D1145 + IBMX (control condition) or 2X GDC in D1145 + IBMX (GDC = 0.5 0.8, 1 mM). Following this, 20 μL cAMP-Glo™ Lysis Buffer was added to each well, and the plate was incubated for 15 min, prior to the addition of PKA and cAMP-Glo™ Reaction Buffer, and the procedure above followed.

### 2.6 GDC treatment of caco2/HT29-MTX monolayers

Caco-2 and HT29-MTX cells were diluted separately in complete DMEM with 1% MEM at 1.2 × 105 cells/mL. A 90:10 cell mix (Caco-2/ HT29-MTX) was added to the apical compartment of 12 well plates containing polyester permeable-membrane inserts (Sarstedt Group) at 6 × 104 cells/well (500 μL). The basolateral compartment received 1.5 mL complete DMEM with 1% MEM. Media was replaced every 2 days and monolayer integrity measured by TEER (Ω × cm^2^, Millicell-ERS Voltohmmeter, Merck Millipore) every 7 days (16 h after media change). To determine the optimal experimental time point for barrier maturity, transepithelial electrical resistance (TEER) was monitored at several intervals (days 7, 14, 21, 24, and 25). Based on these measurements, the monolayer was considered stabilized by day 25, which falls within the widely accepted 21–29 day window for Caco-2 differentiation (59). Senescence markers p21 and p53 were monitored by RT-qPCR (see Supplementary Figure 3) to evaluate cell senescence.

At day 24 monolayers were washed twice and incubated with DMEM D1145 for 16 h. Monolayers with TEER > 1,000 Ω × cm^2^ on day 25 were used. Monolayers were washed twice with HBSS and incubated with DMEM D1145 (apical = 0.4 mL and basolateral = 1.5 mL) for 30 min for acclimatization. GDC (5X in DMEM D1145) was added to the apical compartment (100 μL) to a final concentration of 0.5, 0.8 and 1 mM. Monolayers were incubated for 2 h. TEER was measured at times 0, 1 and 2 h. Control monolayers received DMEM D1145 only. Apical and basolateral samples were collected and stored at −20°C for further analysis. Monolayers were either kept for further experiments or lysed for RT-PCR. TEER values are expressed as Ω × cm^2^- blank (Transwell insert with media in the absence of cells).

### 2.7 Monolayer recovery

On day 25, GDC was removed from Caco-2/HT29-MTX monolayers, which were then washed twice with HBSS. DMEM D1145 was added to the apical (0.5 mL) and basolateral (1.5 mL) compartments, and monolayers were incubated for 24 h. TEER measurements were taken at 1 and 24 h thereafter.

For real-time TEER, Caco-2/HT29-MTX cells (90:10) were seeded at a concentration of 4.8 × 10^5^ cells/well (2 mL/well) in a 6-well plate containing polyester permeable-membrane inserts (basolateral chamber = 3 mL). Media was replaced every 2 days and monolayer integrity tracked by Millicell. On day 24, cells were washed twice with HBSS and incubated for 16 h with DMEM D1145. On day 25, the permeable inserts with monolayers attached were placed into the CellZscope plate (nanoAnalytics GmbH, Germany) for real-time TEER measurements. Only monolayers with a TEER > 1,000 Ω × cm^2^ were used. The monolayers were initially washed with HBSS, then equilibrated for 30 min by adding 1.6 mL DMEM D1145 to the apical side and 4 mL to the basolateral side. Then 0.4 mL 5X GDC in DMEM D1145 was added for 2 h. Monolayers were washed twice with HBSS and incubated for 24 h with fresh DMEM D1145. The CellZscope system measured TEER every h.

### 2.8 Permeability to lactulose

The protocol for measuring monolayer permeability to lactulose was adapted from (37). Lactulose (61360-25G) was prepared in HBSS (2.5 mg/mL) and filter sterilized. GDC treated monolayers were washed twice and acclimatized for 30 min at 37° in HBSS (0.4 mL in the apical and 1.5 mL in the basolateral). Lactulose (0.5 mg/mL in HBSS, 100 μL) was added to the apical side, and monolayers incubated for 4 h at 37°C. TEER was measured at T0 and T4h. Apical and basolateral samples were collected and stored at −20°.

For the quantification, a high performance anion exchange chromatography with pulsed amperometric detection (HPAEC-PAD) on an ICS3000 system equipped with an electrochemical detector (Dionex, USA) was employed. Apical samples were diluted 1 in 100 in Milli-Q H_2_O while the basolateral samples were analyzed undiluted. All samples were filtered with a 0.2 μm nylon syringe filter. Separation was conducted using a CarboPac PA100 column (4 × 250 mm) preceded by a guard column (CarboPac PA-100, 4 × 50 mm) at a flow rate of 1 mL/min. Eluent A = 100 mM NaOH; Eluent B = 100 mM NaOH+500 mM sodium acetate (NaOAc). Elution proceeded according to the following gradient: from t = 0 to 6 min, 100% Eluent A; at t = 13 min, 51.4% Eluent A and 48.6% Eluent B; from t = 13.1 to 23.1 min, equilibrated at 100% Eluent A.

Lactulose levels were quantified by comparing peak areas with known lactulose standards (10 mg/L). The results were expressed as lactulose apparent permeability (Papp) calculated with the following formula:


$$\begin{array}{l} \text{Papp }\left(\text{cm}/\text{min}\right)\;=\left({\text{C}}_{\text{Baso}}\times \text{V}\right)/\left(\text{t}\times \text{A}\times {\text{C}}_{\text{In}}\right) \end{array}$$

Where: C_Baso_ = Concentration of lactulose in the basolateral compartment (mg/L); *V* = Volume of the basolateral compartment (L); *t* = incubation time (240 min); *A* = surface area (1.1 cm^2^); C_In_ = initial concentration in the apical (mg/L).

### 2.9 Lactate dehydrogenase (LDH) assay

Briefly, 50 μL from the apical chamber of GDC-treated monolayers were transferred to a 96-well flat-bottom plate, and mixed with equal volume of LDH Cytotoxicity Assay reaction mixture. The plate was incubated at room temperature in the dark for 30 min to allow LDH conversion of lactate to pyruvate, initiating a reduction of formazan dye. After incubation, 50 μL stop solution was added to each well, and absorbance was measured at 490 nm and 680 nm using a Synergy HT plate reader (Biotek). Cytotoxicity was then calculated as follows:


$$\begin{array}{l} \%\text{ cytotoxicity}=\;(\text{GDC}-\text{treated LDH activity}\\ \;-\text{Spontaneous LDH activity})/(\text{Maxium LDH}\\ \text{ activity }-\text{Spontaneous LDH activity})\;\times \text{100} \end{array}$$

Where: GDC-treated LDH activity = GDC treated monolayer supernatant; Spontaneous LDH activity = supernatant from control monolayers, Maximum LDH activity = LDH released from 50 μL of cell lysate (control monolayers lysed, with 500 μL of 1% TritonX, by pipetting).

### 2.10 Alkaline phosphatase activity

Caco-2/HT29-MTX monolayers were tested for alkaline phosphatase activity after a 2 h treatment with 0, 0.5, 0.8, 1 mM GDC. In each well, the apical fluid was collected and the monolayer was then washed twice with PBS. Monolayers were lysed with 0.2% TritonX (0.5 mL) in Milli-Q H_2_O by shaking for 20 min at room temperature. Then 50 μL apical sample or 50 μL cell lysate were transferred into a clear flat-bottom 96-well plate. The activity of alkaline phosphatase was measured with the Alkaline Phosphatase Assay kit (MAK447), in which p-nitrophenyl phosphate is converted into p-nitrophenol and inorganic phosphate. After 4 min of reaction, absorbance was measured at 405 nm using a Synergy HT plate reader. The activity of alkaline phosphatase was calculated with the following formula:


$$\begin{array}{l} \left[\left(\text{O}{\text{D}}_{\text{T}4}-\text{ O}{\text{D}}_{\text{T}0}\right)\times \text{RxnVol}\right]/\text{εPNP}\times \text{L}\times \text{SmplVol}\times \text{t}; \end{array}$$

Where: OD_T0_ = D value at 405 nm at time 0, OD_T0_ = OD value at 405 nm at time 4 min, RxnVol = reaction volume, εPNP = absorptivity coefficient for p-Nitrophenol (18.75 mM^−1^× cm^−1^), *L* = light path in cm, SmplVol = sample volume, *t* = reaction time, 4 min. The light path (*L*) has been calculated with the following formula:


$$\begin{array}{l} L=\left({\text{OD}}_{\text{Cal}}-{\text{OD}}_{\text{Blank}}\right)/\left(\text{εC}\right); \end{array}$$

Where: OD_Cal_ = absorbance at 405 nm of the Calibrator of the kit, OD_Blank_ = absorbance at 405 nm of the blank (well with just media and no cells), ε = molar absorptivity coefficient, C = path length of the plate in cm.

### 2.11 Cytokine quantification

For the pro-inflammatory cytokines (IL-8, IL-6 and TNF), apical samples were centrifuged at 900 g for 5 min to remove debris. The analysis was performed with 25 μL supernatant using the Human Milliplex Map Kit (HSTCMAG-28SK, Merck Millipore) and MagPix fluorescent detection system (Luminex, The Netherlands), according to the manufacturer's instructions. Milliplex assay detects IL-6 in the concentration range 0.18–750 pg/mL, IL-8 between 0.31 and 1,250 pg/mL and TNF between 0.43 and 1,750 pg/mL.

### 2.12 OCLN immunofluorescence

After GDC treatment, monolayers were washed twice with PBS and fixed for 30 min at room temperature by adding 500 μL 75% ethanol in PBS to the apical chamber. Monolayers were washed twice with PBS and kept at 4°C in PBS until use. Monolayers were permeabilized with 75% acetone in PBS for 3 min at room temperature, washed three times with PBS and incubated for 30 min at room temperature with the blocking solution (1% BSA in PBS). The blocking solution was removed and 150 μL primary antibody (71–1,500, Rabbit Occludin Polyclonal Antibody, ThermoFisher Scientific) diluted in blocking solution (8.33 μg/mL) was added. Monolayers were incubated overnight at 4°C. The following day, monolayers were washed twice with PBS and 150 μL secondary antibody (A-21206, Alexafluor 488 Donkey anti-rabbit, ThermoFisher Scientific) in blocking solution was added (11 μg/mL). Monolayers were incubated for 1 h in the dark at room temperature, washed three times with MilliQ H_2_O and incubated with DAPI (62,248, ThermoFisher Scientific; 1:500 dilution in MilliQ H_2_O) for 2 min, in the dark, at room temperature. The permeable supports of the Transwell plates were excised with a scalpel and mounted on a microscope slide with 40 μL mounting media (P36930, ProLong™ Gold Antifade Mountant, ThermoFisher Scientific).

Microscopy images were captured with Leica TCS SP5 Confocal Laser Scanning Microscope with 405 Diode and Argon lasers (Leica Microsystems, Wetzlar, Germany) and HC PL APO 63x/1,40 OIL CS2 objective. Pinhole size was 85.65 nm. Images were captured with the following settings: scan mode—xyz, format—1,024 × 1,024, speed—440 Hz, pixel size—240.50 x 240.50 x 713.35 nm, line and frame average—6, z-step size—0.71 μm. Alexa Fluor 488 acquisition parameters were: 520–560 nm (emission range), 929 V (gain) and 100% (laser intensity). DAPI acquisition parameters were: 410–480 (emission range), 700 V (gain) and 64% (laser intensity). Images were acquired using the Leica Application Suite AV (v 2.7.3.9723) software (Leica Microsystems). Fiji/ImageJ software (version 1.54f, Java 1.8.0_322) was used for subsequent analysis of OCLN fluoresence intensity. Regions of Interest (ROIs) were manually selected from acquired images based on where the nuclei were well visible. For intensity quantification, the z-plane corresponding to the middle of the nuclei was chosen to ensure consistent measurement across samples. Intensity threshold was determined with the Otsu algorithm. The OCLN fluorescence intensity was calculated as raw integrated density (Raw IntDen), which represents the sum of pixel intensity within each ROI, normalized by the number of nuclei. For improved visualization, contrast adjustments were applied to the images using ImageJ software. The adjustments were performed uniformly across all images.

### 2.13 mRNA expression by real time PCR

Cell monolayers treated with GDC were lysed by the addition of RLT buffer (350 μL) with 1% β-mercaptoethanol on ice for 5 min. Cell lysate was collected and stored at −80°C prior to use. Total RNA was extracted with the RNeasy Mini Kit with DNase digestion (Qiagen), following manufacturer's instructions. RNA was quantified with Nanodrop 1,000 (Thermo Fisher Scientific) and only the samples meeting the criteria of OD_260/280_ ratios between 1.8 and 2.0 were used for cDNA synthesis. Reverse transcription from 0.5 μg of total RNA to cDNA was conducted using the SensiFAST cDNA Synthesis kit (Bioline, UK). mRNA transcripts were quantified using LightCycler 480 SYBR Green I Master kit (Roche Diagnostics Ltd., UK) using a LightCycler 96 instrument.

Primer sequences and annealing temperatures, for Claudin-2 (CLDN-2), Junctional Adhesion Molecule 1 (JAM-1), Occludin (OCLN), Zonula Occludens-1 (ZO-1), Claudin-4 (CLDN-4), Ribosomal Protein Lateral Stalk Subunit (P0-RPLP0), Mucin-1 (MUC-1), Mucin-2 (MUC-2), Mucin-5AC (MUC-5A) cyclin-dependent kinase inhibitor 1 (p21) and tumor protein p53 (p53), are listed in Supplementary Table 1. The reaction mixture for each well consisted of 5 μL of master mix, 0.5 μL of each forward and reverse primer (10 μM) and 1 μL of 1 in 10 dilution of cDNA.

The 2^−ΔΔCt^ method was employed to calculate the relative amount of the target gene, where ΔΔCt = (Ct _Target gene_ – Ct _RPLP0_) _GDC test_ – (Ct _Target gene_ – Ct _RPLP0_) _Control_.

Here, Ct = theshold cycle for either the target gene or RPLP0; test = monolayers treated with 0.5, 0.8 or 1 mM GDC; control = DMEM D1145 media treated monolayers.

### 2.14 Mucus quantification by Alcian blue

GDC-treated monolayers were washed twice with PBS. Then, 0.5 mL of 1% Alcian blue (A5268) in 0.16 M sucrose + 0.05 M sodium acetate at pH 5.8 was added to the apical compartment and incubated at 37°C for 30 min. After incubation, the stain was removed and monolayers were washed, at least 3 times, with PBS (until the PBS was clear). Monolayers were then lysed by pipetting with 0.5 mL RIPA buffer with 1% EDTA. Collected cell lysates were briefly spun to remove debris. The supernatant (150 μL) was placed in a clear flat-bottom 96-well plate in duplicate and absorbance measured at 620 nm in a Biotek Synergy HT plate reader. Results are presented as the absorbance at 620 nm–blank (RIPA buffer with 1% EDTA alone).

### 2.15 Gastrointestinal digestion of infant milk formula (IMF)

Infant milk formula (IMF) was reconstituted following the manufacturer's instructions by dissolving 4.3 g of powder in 30 mL of MilliQ H_2_O. The infant simulated gastro-intestinal digestion (SGID) was performed as per Ménard et al. (84). The protocol was adjusted to ensure full activation of pepsin and trypsin, with a specific focus on protein digestion. As a result, lipase activity was not prioritized in this setup. This approach aligns with INFOGEST recommendations, which support adapting digestion parameters according to the nutrient class being investigated (60). A volume of 5 mL of reconstituted IMF was mixed with 2.94 mL infant simulated gastric fluids containing 268 U/mL RGE 15 (pepsin activity = 596 U/mg). The pH was brought to 5.3 with 1 M HCl. Samples were incubated for 1 h at 37°C in a shaking incubator (100 rpm). To stop the gastric phase and inhibit pepsin activity, the pH was increased to 7 with 1 M NaOH. Then, 4.88 mL of simulated intestinal fluids containing 16 U/mL pancreatin (lot number SLCM8903, trypsin activity = 8.9 U/mg) and 3.1 mM bovine bile (lot number SLBV1780, bile salts concentration = 440 g/mol) were added. The pH was decreased to 6.6 with 1 M HCl. The samples were incubated again for 1 h at 37°C in a shaking incubator. Following the intestinal phase, digestion was stopped by adding the protease inhibitor 4-(2-aminoethyl) benzene sulfonyl fluoride hydrochloride, dissolved in MilliQ H_2_O at a final concentration of 1 mM, along with Orlistat dissolved in ethanol at a final concentration of 0.1 mM. An SGID control sample, where MilliQ H_2_O replaced the IMF, was subjected to gastrointestinal digestion followed by enzyme inactivation (SGID-H_2_O). The SGID samples were stored at −80°C until use. The protein content of the digested IMF was determined by Pierce BCA Protein Assay Kit (ThermoFisher Scientific).

### 2.16 Caco-2/HT29-MTX monolayers exposure to digested IMF

For the monolayer exposure, 0 mM GDC or 0.8 mM GDC-treated Caco-2/HT29-MTX monolayers were washed twice and incubated with HBSS (0.4 mL in the apical and 1.5 mL in the basolateral side) for 15 min at 37°C for acclimatization. The SGID IMF was defrosted at room temperature, diluted 1 in 3 in HBSS, filtered (0.45 μm PES syringe filters, low protein bind, Sarstedt) and pre-warmed to 37°C. Subsequently, 100 μL SGID IMF or HBSS (buffer only control) was added to the apical compartment (final dilution 1 in 15) for 2 h at 37°C. TEER values were recorded at T0h (after HBSS wash and acclimatization) and at T2h (after 2 h incubation with the digesta). The protein content of the sample applied to the apical side (μg/cm^2^) was calculated as follows: protein content of digesta (BCA assay, μg/mL)/ dilution factor [15]/volume on apical side (0.5 mL)/ Transwell filter area (1.1 cm^2^). Apical and basolateral samples were collected and stored at −20°C for amino acids analysis.

### 2.17 Quantification of the total amino acid in the basolateral chamber

Basolateral samples were defrosted at room temperature, and 0.5 mL of each technical replicate was pooled. A total volume of 1 mL from this pooled sample was transferred to a glass vial, evaporated using a SpeedVac concentrator, and then re-suspended in 100 μL of hydrolysis solution (6 M HCl). Hydrolysis was conducted for 15 h at 110°C. To quantify the derivative forms of cysteine (XCys), cysteine standards (10 and 1 mmol/L) were also hydrolyzed. Prior to hydrolysis, an internal standard of Norvaline was added to each sample to achieve a final concentration of 250 ρmol/L. After hydrolysis, 20 μL of each sample was neutralized by the addition of 20 μL of 6 M NaOH and 20 μL of 0.2 mM HCl, and then filtered using a 0.45 μm nylon syringe filter. The samples and standards (Amino Acid Hydrolysate Standard, Waters Corporation) were derivatized using the AccQ-Tag Ultra Derivatization Kit (Waters Corporation). Each derivatization reaction consisted of mixing 10 μL of the sample with 70 μL of AccQ•Tag Ultra Borate Buffer and 20 μL of AccQ•Tag Ultra reagent, followed by incubation at 55 °C for 10 min. Amino acids detection was performed with ACQUITY Ultra Performance LC^®^ (UPLC^®^) equipped with tunable ultraviolet/visible (TUV) detector (Waters Corporation). Separation was performed with AccQ•Tag Ultra Amino Acid Analysis Column (Eluent 1 = AccQ•Tag Ultra Eluent, Eluent 2 = AccQ•Tag Ultra Eluent B, Waters Corporation; flow rate = 0.7 mL/min; injection volume = 1 μl; column temperature = 55°C). The quantification was performed based on a standard three-point curve. The Empower software (version 3.7.0, Waters Corporation) was used to acquire, process, report, and manage chomatographic information.

### 2.18 Statistical analysis

All experiments were conducted in triplicates or quadruplicate across three separate days, each with technical duplicates or triplicates. Results are expressed as means ± SEM. Statistical analyses were performed using GraphPad Prism 9.1.0 (GraphPad Software, USA) via two-way ANOVA with Tukey's multiple comparison or Dunnet's multiple comparison. Statistical significance was determined at a *P*-value < 0.05.

## 3 Results

### 3.1 IC_50_ of GDC on Caco-2/HT29-MTX is 2.49 mM

The IC_50_ of GDC on the viability of undifferentiated Caco-2/HT29-MTX cells was determined by MTS and non-linear regression analysis (Supplementary Figure 1). The IC_50_ was 2.49 mM, (95% CI = 2.21-3.04 mM). Cell viability was not decreased by GDC concentrations of 0.5, 0.8 and 1 mM GDC compared to the control (cells in DMEM D1145 only; *P* > 0.05). Cells treated with 1% Triton X served as a positive control, showing a viability of 0.457 ± 0.037%.

### 3.2 GDC decreases TEER with subsequent recovery

To assess the effect of non-cytotoxic GDC on the intestinal barrier integrity, 25-day old differentiated Caco-2/HT29-MTX 90:10 monolayers were exposed for 2 h to 0.5, 0.8 and 1 mM GDC. Initially, development of the barrier overtime was tracked by TEER measurements at 7, 14, 21, 24 and 25 days after seeding (Supplementary Figure 2). At day 21 TEER values were 848 ± 36.54 Ω × cm^2^. It took a further 3 days for the TEER values to stabilize. By day 24, TEER values had increased to 1261 ± 34.67 Ω × cm^2^ and had stabilized, with no significant increase from day 24 to day 25 (1302.81 ± 35.162 Ω × cm^2^, *P* > 0.05) (Supplementary Figure 2). Although within the range (day 21-29) recommended by Hubatsch et al. (59), day 25 monolayers were checked for senescence, by quantifying mRNA transcript levels of senescence markers p21 and p53 (61). Transcript levels of p21 and p53 did not significantly change in day 25 monolayers compared to day 21 (*P* > 0.05; Supplementary Figure 3).

**Figure 1:**
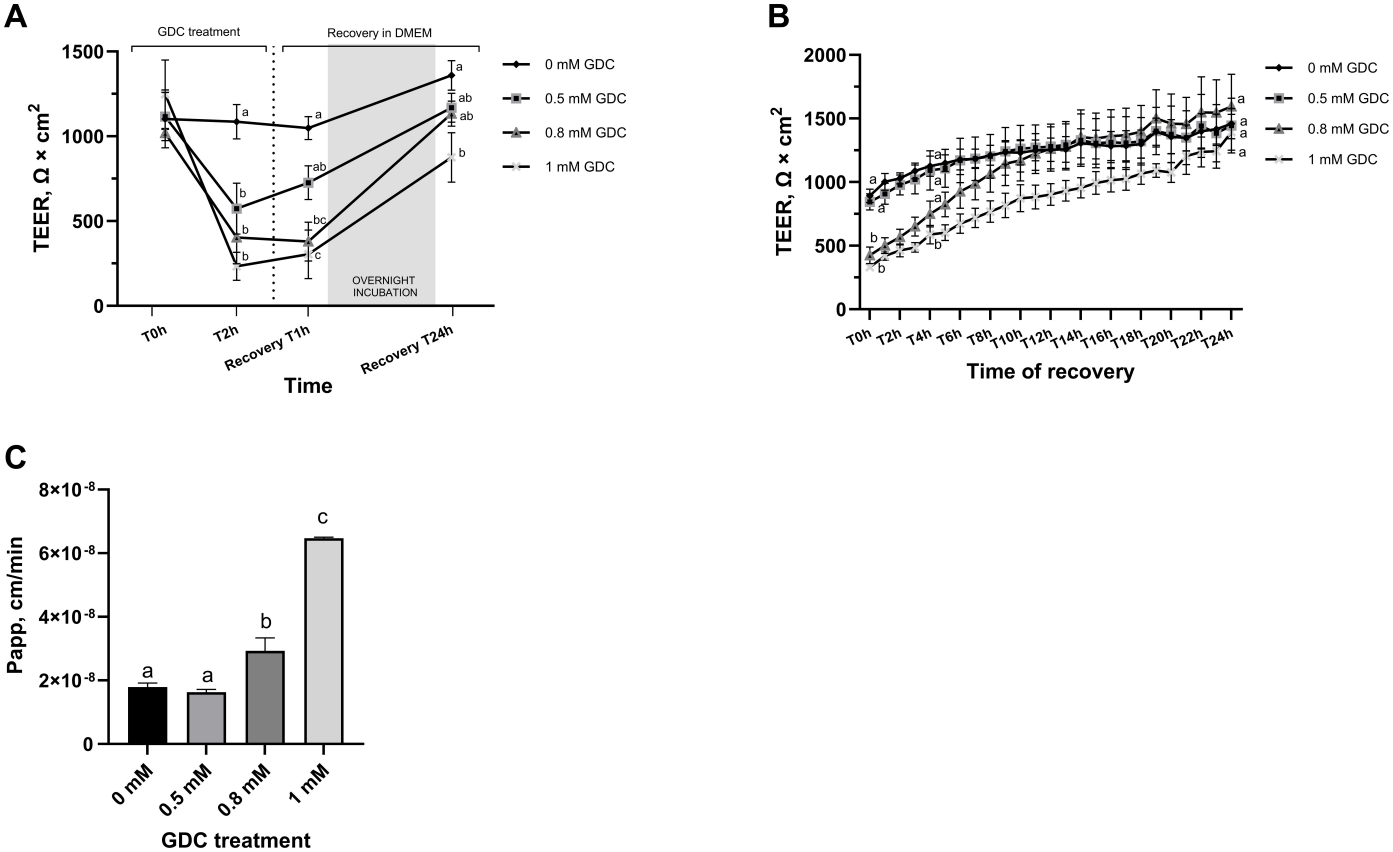
**(A)** Trans-Epithelial Electrical Resistance (TEER) and **(B)** lactulose permeability of GDC-treated Caco-2/HT29-MTX monolayers. TEER value at day 25 = 1,302.81 ± 70.32 Ω × cm^2^. Caco-2/HT29-MTX 90:10 monolayers were treated for 2 h (T2h) with 0.5, 0.8 or 1 mM GDC in DMEM D1145. **(A)** GDC treatment with TEER values recorded by Millicell ERS Voltohmmeter. T0 = monolayers washed twice with HBSS and GDC added, T2h = GDC treatment for 2 h, Recovery T1h–T24h = timeline of recovery phase in DMEM D1145 alone. **(B)** Recovery phase (T0–T24h) with TEER values recorded by CellZscope automated TEER monitoring system. **(C)** Lactulose apparent permeability (Papp; cm/min). GDC treated monolayer were washed and treated with lactulose for 4 h. Results are presented as biological triplicates with technical duplicate ± SEM. Statistical difference was assessed **(A, B)** between treatments at a time point by two-way ANOVA or **(C)** with one-way ANOVA with Tukey's multiple comparison. Different letters signify statistical difference (*P* < 0.05).

Once day 25 monolayer were treated with GDC, all 3 GDC concentrations resulted in significantly lower TEER values (Figure 1A, *P* < 0.05) at 2 h compared to monolayers in media alone (0 mM GDC = 1,086.071 ± 01.28; 0.5 mM GDC = 573.34 ± 149.69; 0.8 mM GDC = 403.15 ± 155.29; 1 mM GDC = 233.26 ± 82.20 Ω × cm^2^). The calculated fold decreases in TEER relative to the control were as follows: 0.5 mM GDC = 1.89, 0.8 mM GDC = 2.69, 1 mM GDC = 4.65. Once GDC was removed (recovery phase), monolayers treated with 0.5 mM GDC had similar TEER values to values of control monolayers within 1 h (*P* > 0.05). Monolayers treated with 0.8 or 1 mM GDC were still significantly lower than controls at 1 h (*P* < 0.0001). By 24 h in the recovery phase, 0.5 and 0.8 mM GDC monolayers had TEER value increases similar to control monolayers (0 mM GDC = 1,359.05 ± 86.79; 0.5 mM = 1,168.2 ± 85.35; 0.8 mM = 1,133.55 ± 73.76 Ω × cm^2^; *P* > 0.05), while 1 mM GDC was still struggling to recover (1 mM = 875.29 ± 145.41 Ω × cm^2^, *P* < 0.01).

This recovery is critical to simulate the transient nature of neonatal gut permeability and to enable post-treatment applications, such as digesta exposure or intervention studies that might induce gut barrier restoration. To examine the recovery phase in detail, TEER values were measured every hour for 24 h using a CellZscope real-time cell monitoring system (Figure 1B). At the start of the recovery phase (T0h), TEER values for monolayers treated with 0.8 mM and 1 mM GDC were significantly lower than those of the control monolayers (*P* < 0.05). CellZcope measurements revealed that monolayers treated with 0.5 mM GDC showed no significant difference from 0 mM GDC at the beginning of the recovery phase. TEER values of 0.8 mM GDC treated monolayers reached levels similar to control monolayers by 4 h of recovery (T4h; *P* > 0.05). By the end of the 24-h recovery period (T24h), TEER values for all GDC-treated monolayers had returned to levels comparable to those of the control condition (0 mM GDC, *P* > 0.05).

### 3.3 GDC-treated monolayers have a higher permeability to lactulose

To evaluate whether decreased TEER values in GDC-treated monolayers corresponded to increased permeability, a lactulose permeability assay was performed on these monolayers (Figure 1C). Caco-2/HT29-MTX monolayers treated with 0.8 mM and 1 mM GDC had a significantly higher lactulose Papp (0.8 mM GDC = *P* < 0.05, 1 mM GDC = *P* < 0.001) compared to 0 mM GDC. The lactulose Papp increased by 0.91-fold for 0.5 mM GDC, 1.64-fold for 0.8 mM GDC and 3.61-fold for 1 mM GDC (Papp 0 mM GDC = 1.79 × 10^−8^ cm/s ± 1.21 × 10^−9^). Mannitol was not included in the permeability assessment, as mannitol is transported paracellularly and transcellularly *in vivo*, while only paracellularly *in vitro* (62).

### 3.4 GDC treatment does not cause monolayer cytotoxicity

**Figure 2:**
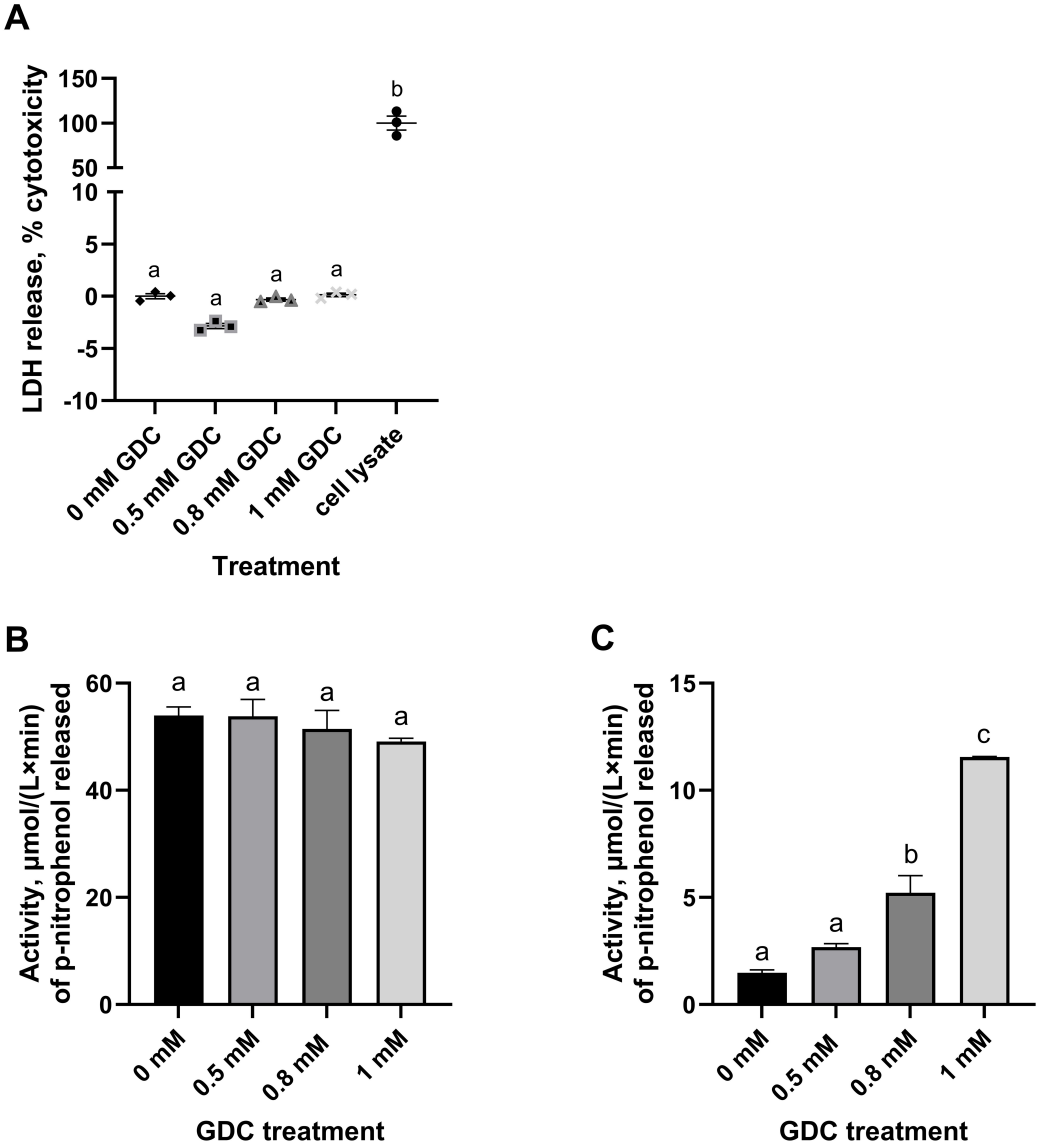
Effect of GDC treatment on **(A)** LDH release, **(B)** intracellular and **(C)** extracellular alkaline phosphatase activity in Caco-2/HT29-MTX monolayers. Caco-2/HT29-MTX 90:10 monolayers were grown for 25 days and exposed for 2 h to 0.5, 0.8 or 1 mM GDC. **(A)** % of cytotoxicity measured by the release of LDH in the apical compartment, given by the amount of formazan produced in the enzymatic reaction with the assay mixture. LDH was 0 mM GDC = monolayers in DMEM 1,145 only (set as 0% cytotoxicity); Cell Lysate = positive control, cells lysed with 1% Triton-X (set as 100% cytotoxicity). **(B)** Intracellular (cell lysate) and **(C)** extracellular (apical supernatant) alkaline phosphatase activity was measured using p-nitrophenyl phosphate as substrate. Alkaline phosphatase activity was calculated as μmol/(L x min) of p-nitrophenol released. Results are presented as the average of a biological triplicate and a technical duplicate ± SEM. Statistical difference in the treatments was assessed by one-way ANOVA with Tukey's multiple comparison and is indicated with different letters (*P* < 0.05).

LDH release was measured in the GDC treated monolayers, as LDH leakage indicates cytoplasmic damage. None of the GDC concentrations increased the LDH release, suggesting GDC is not causing cytoplasmic leakage (Figure 2A; *P* > 0.05).

### 3.5 GDC increases extracellular, but not intracellular, alkaline phosphatase activity

To study the effects of GDC on epithelial function, the intracellular and extracellular alkaline phosphatase activity were measured in GDC treated Caco-2/HT29-MTX monolayers (Figures 2B, C). No significant difference was found in the intracellular alkaline phosphatase activity of the GDC treated monolayers compared to the 0 mM GDC (53.99 ± 1.59 μmol/(L x min); *P* > 0.05). However, the 0.8 and 1 mM GDC-treated monolayers showed a significantly increased extracellular activity (*P* < 0.05 and *P* < 0.001) compared to untreated (Figure 2C). The extracellular alkaline phosphatase activity was: 0 mM GDC = 1.478 ± 0.13, 0.8 mM GDC = 5.219 ± 0.79, 1 mM GDC = 11.55 ± 0.02 μmol/(L x min).

### 3.6 GDC treatment does not cause monolayer inflammation

To investigate whether or not GDC treatment resulted in an inflammatory response, cytokines IL-8, IL-6 and TNF were tracked in the apical compartment of GDC-treated monolayers. These cytokines were chosen as they activate the key inflammation pathways such as NF-kB and ERK-MAPK (63) and are secreted by Caco-2 monolayers under inflammatory conditions (63, 64). Secreted IL-6 and TNF could not be detected in the 0 mM GDC nor the GDC-treated monolayers. IL-8 was detected but levels did not significantly differ with GDC treatment (0 mM GDC = 5.84 ± 0.59; 0.5 mM GDC = 9.54 ± 4.21; 0.8 mM GDC = 8.33 ± 2.81; 1 mM GDC = 11.32 ± 3.81 ρg/mL).

### 3.7 GDC reduces OCLN fluorescence intensity and modulates tight junction proteins mRNA expression

**Figure 3:**
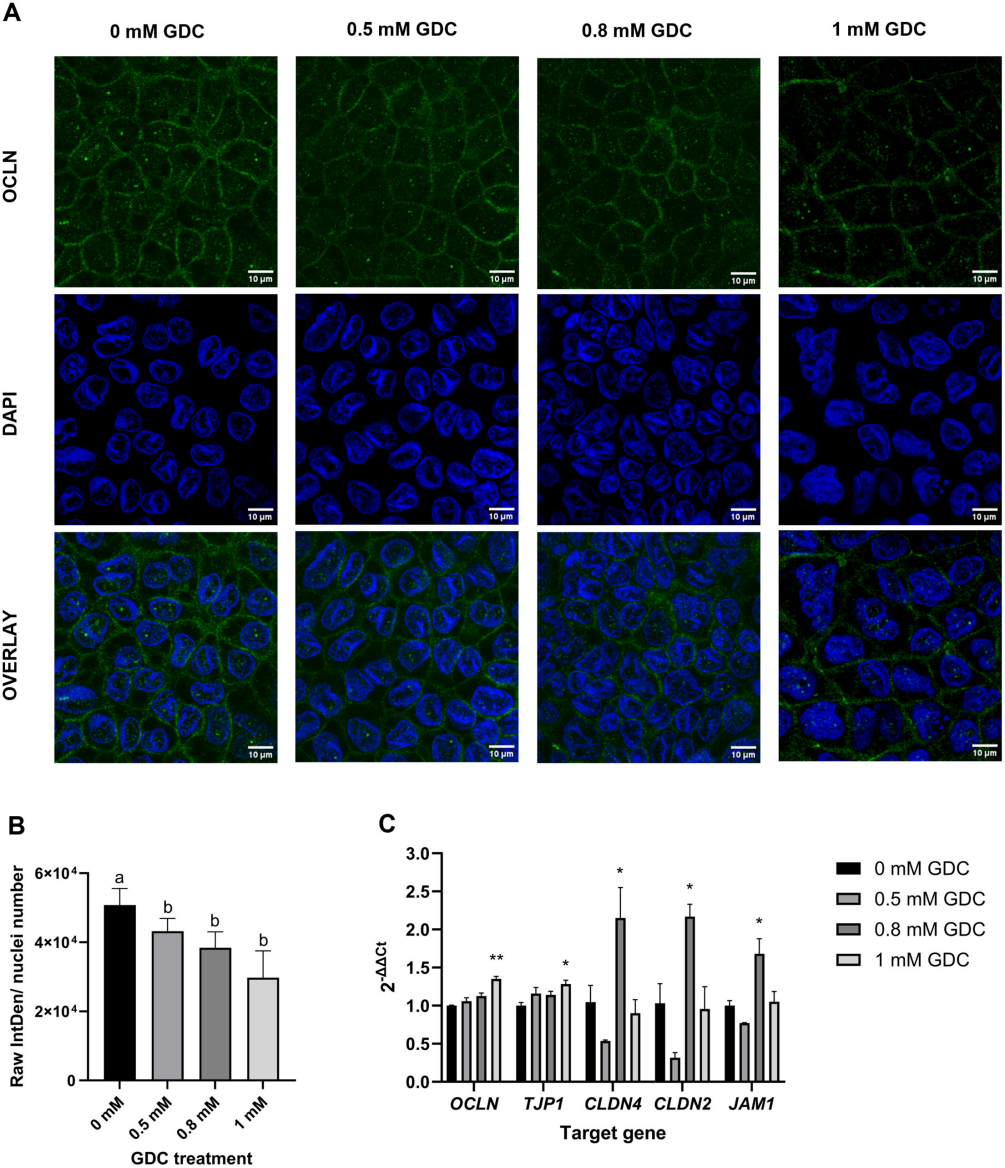
Effect of GDC treatment on **(A, B)** occludin (OCLN) localization and **(C)** tight junction protein expression in Caco-2/HT29-MTX monolayers. Caco-2/ HT29-MTX 90:10 monolayers were grown for 25 days and treated for 2 h with 0.5, 0.8 and 1 mM GDC. **(A)** Representative confocal microscopy images of monolayers stained with OCLN (Alexa488, green) and nuclei stained with DAPI (blue). Scale bar = 10 μm. OCLN was White arrows indicate OCLN at the cell membrane. **(B)** Fluorescence intensity of OCLN from images using the Otsu thresholding algorithm in Fiji-ImageJ and normalized to the number of nuclei. Results represent the average of three biological replicates and three images for each replicate (mean ± SEM). Statistical differences (*P* < 0.05) between treatments was evaluated using one-way ANOVA and indicated by different letters. 0 mM GDC = monolayers in DMEM 1,145 media only. **(C)** Relative mRNA expression levels of tight junction proteins *OCLN*, zonulin (ZO-1, *TJP1*), claudin-4 (*CLDN4*), claudin-2 (*CLDN2*), and junctional adhesion molecule 1 (*JAM1*) in monolayers treated with GDC. Transcript levels were normalized to the housekeeping gene *RPLP0* mRNA and monolayers in DMEM 1,145 alone (0 mM GDC), employing the 2^−ΔΔCt^ method. Results represent the average of three biological and four technical replicates, presented as mean ± SEM. Statistical significance was evaluated using one-way ANOVA with Dunnett's multiple comparison test (treatment vs. control). Asterisks denote statistical difference between each treatment and 0 mM GDC (*P* < 0.05).

To determine the effect of GDC on the gut barrier integrity, OCLN immunofluorescence was performed on the monolayers treated with 0, 0.5, 0.8 and 1 mM GDC (Figures 3A, B). The results revealed a significantly lower fluorescence intensity of OCLN per number of cell nuclei for all GDC treatments (Figure 3B; *P* < 0.05). Specifically, OCLN intensity for 0 mM GDC was 50,758 ± 4,809, for 0.5 mM GDC was 43,214 ± 3,716 (85.28 ± 0.89% of control), for 0.8 mM GDC was 38,447 ± 4,597 (75.79 ± 5.02% of control) and for 1 mM GDC was 29,783 ± 7,740 (57.40 ± 10.26% of control).

To assess if the lower protein levels was due to lower mRNA transcripts, *OCLN* mRNA and associated tight junction biomarkers (*ZO-1, CLDN-2/4* and *JAM-1*) were measured in GDC-treated Caco-2/HT29-MTX monolayers (Figure 3C). *OCLN* and *ZO-1* mRNA levels were significantly higher in 1 mM GDC-treated monolayers (*OCLN* = *P* < 0.01; *ZO-1* = *P* < 0.05) compared to 0 mM GDC, whilst treatment of monolayers with 0.5 or 0.8 mM GDC did not alter *OCLN* or *ZO-1* mRNA transcript levels (*P* > 0.05). *CLDN-2, CLDN-4* and *JAM-1* expression was increased in 0.8 mM GDC –treated monolayers (*P* < 0.05), compared to 0 mM GDC.

### 3.8 GDC increases mucus production but not mucins expression

To determine whether GDC treatment affects the mucus layer, GDC-treated monolayers were stained with Alcian blue to quantify acidic mucins. Acidic mucins were selected as the main focus because the neonatal intestinal mucus is enriched in these glycoproteins, particularly sialomucins and sulfomucins, which predominate at birth and play critical roles in early-life gut barrier function and host–microbiota interactions (27).

**Figure 4:**
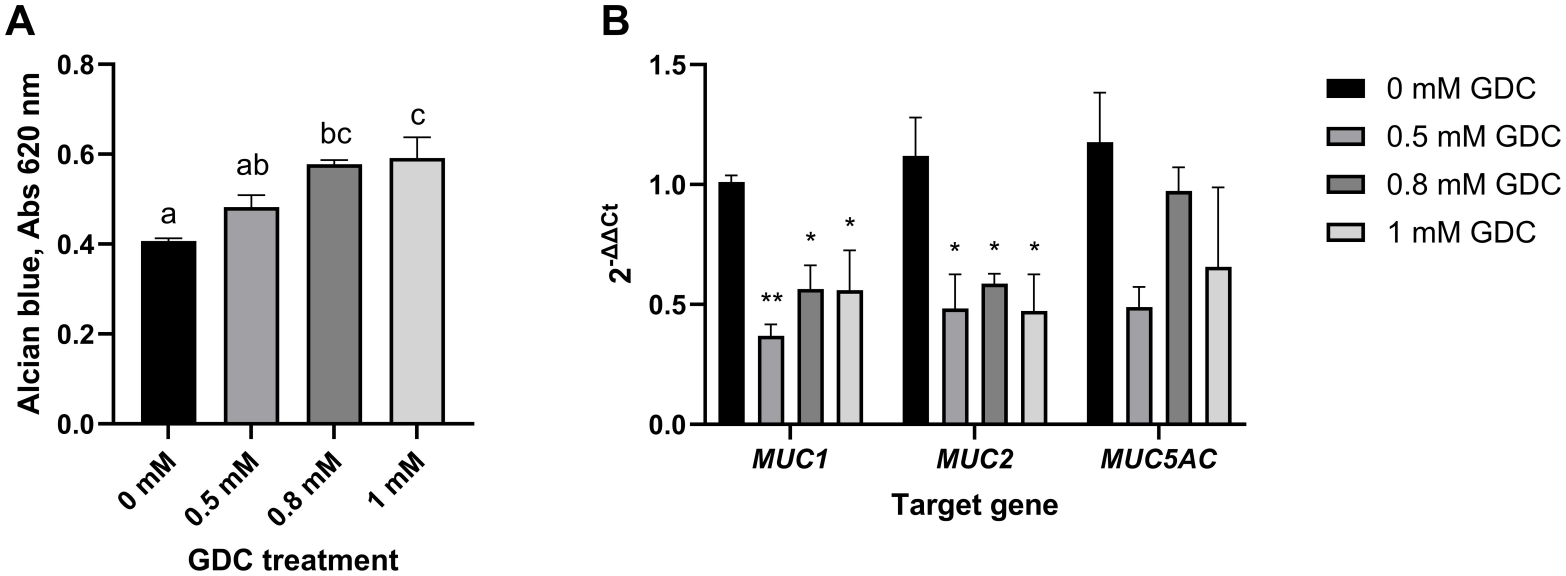
**(A)** Mucus staining **(B)** and mucins mRNA expression in Caco-2/HT29-MTX monolayers treated with GDC. **(A)** 25-day-old Caco-2/HT29-MTX 90:10 monolayers treated with 0.5, 0.8 and 1 mM GDC for 2 h were stained with Alcian Blue, lysed and absorbance at 490 nm recorded. **(B)** mRNA expression of mucin-1 (*MUC1*), mucin-2 (*MUC2*) and mucin-5AC (*MUC5AC*). Transcript levels were determined using RT-PCR and normalized to the housekeeping gene RPLP0 mRNA and monolayers in media alone (0 mM GDC), employing the 2^−ΔΔCt^ method. Results represent the average of three biological and four technical replicates, presented as mean ± SEM. Statistical significance (*P* < 0.05) was evaluated using one-way ANOVA with **(A)** Dunnet's multiple comparison test (treatment vs. control) or **(B)** Tukey's multiple comparison. **(A)** Different letters denote statistical difference between each condition. **(B)** Asterisks denote statistical difference between each treatment and the control within each gene.

As shown in Figure 4A, 0.8 mM and 1 mM GDC-treated cells exhibited a significant increase in Alcian blue staining intensity compared to 0 mM (P < 0.05). Monolayers treated with 0.5 mM GDC had similar Alcian blue staining 0 mM and 0.8 mM GDC-treated monolayers (*P* > 0.05).

The expression of MUC-1, MUC-2, and MUC-5AC was assessed using RT-qPCR (Figure 4B). The analysis revealed that GDC-treated monolayers had a significantly lower mRNA expression of MUC-1 (0.5 mM GDC = *P* < 0.01, 0.8/ 1 mM = *P* < 0.05) and MUC-2 (*P* < 0.05) compared to 0 mM GDC, with no difference in MUC-5AC.

### 3.9 GDC does not activate the apoptotic cascade but decreases cAMP levels

**Figure 5:**
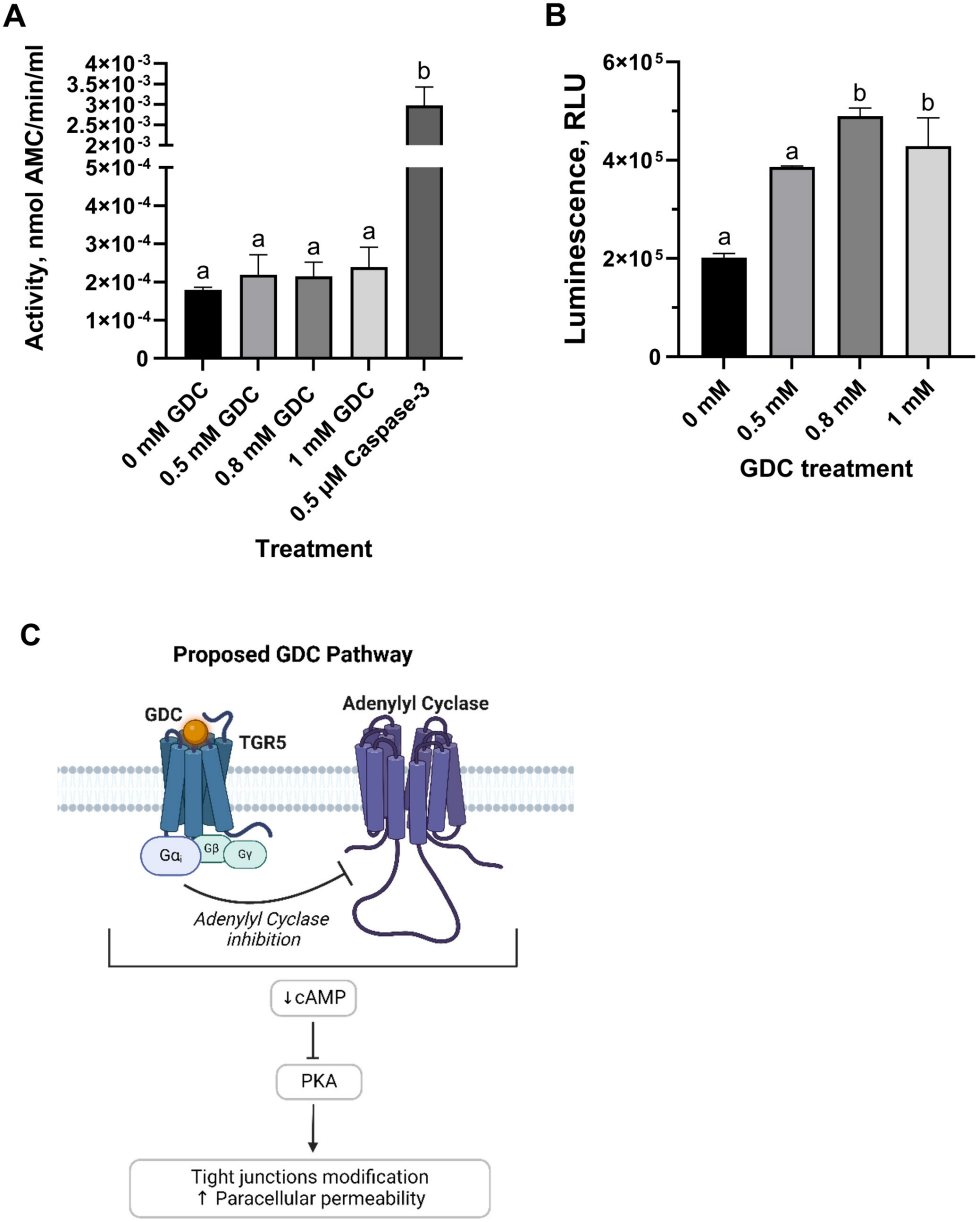
**(A)** Caspase-3 activity and **(B)** intracellular cAMP levels in undifferentiated Caco-2/HT29-MTX cells treated with GDC and **(C)** proposed mechanism of action. Caco-2/HT29-MTX 90:10 cells were seeded at 10^5^ cells/well, grown for 24 h and then exposed for 2 h to 0.5, 0.8 or 1 mM GDC. **(A)** Caspase-3 activity, reported as nmol of 7-Amino-4-methylcoumarin—AMC—released per min per mL of cell lysate, was measured using acetyl-Asp-Glu-Val-Asp-7-amido-4-methylcoumarin as the reaction substrate. **(B)** Intracellular cAMP was measured indirectly via protein kinase A activity, where cAMP activates protein kinase A, reducing ATP and thereby decreasing light output in a coupled luciferase reaction. 0 mM GDC = monolayers in DMEM D1145 + IBMX. Results are presented as Relative Light Unit (RLU), which inversely correlates with cAMP levels. 0 mM GDC RLU = 201,707 ± 8,486. The results represent the average of a biological triplicate and a technical duplicate ± SEM. Statistical difference in the treatments was assessed by one-way ANOVA with Tukey's multiple comparison and is indicated with different letters (*P* < 0.05). **(C)** Proposed mechanism of action of GDC. By binding to the G-protein coupled receptor TGR5 (Takeda G protein-coupled Receptor), GDC inhibits adenylate cyclase via the Gαi subunit, leading to reduced cAMP levels and PKA inactivation. Figure 5c was created with BioRender.com.

To investigate the mechanism of action of this bile salt, Caspase 3 activity assay was performed on undifferentiated Caco-2/HT29-MTX cells, as an indicator of apoptotic pathway activation. No statistical difference was found between GDC treated cells compared to cells in media overnight (*P* > 0.05; Figure 5A).

Whether or not GDC treatment results in modulation of intracellular cAMP levels, cAMP was measured in undifferentiated Caco-2/HT29-MTX cells following treatment with 0, 0.5, 0.8 and 1 mM GDC (Figure 5B). Cells treated with 0.8 and 1 mM GDC resulted in a significantly higher Relative Luminescence Unit (RLU; which equates to lower cAMP levels) compared to control cells without GDC (0 mM GDC = DMEM D1145 + IBMX only; *P* < 0.05). No statistical difference was found between, control and 0.5 mM GDC treated cells (*P* > 0.05). To determine if GDC inhibits PKA activity directly, GDC solutions were mixed with cAMP and prior to PKA activity measurement. There was no significant difference (*P* > 0.05) between media only and GDC solutions, indicating that GDC does not inhibit PKA activity (Supplementary Figure 4).

### 3.10 GDC (0.8 mM) increases amino acids transport after exposure to digested IMF

**Figure 6:**
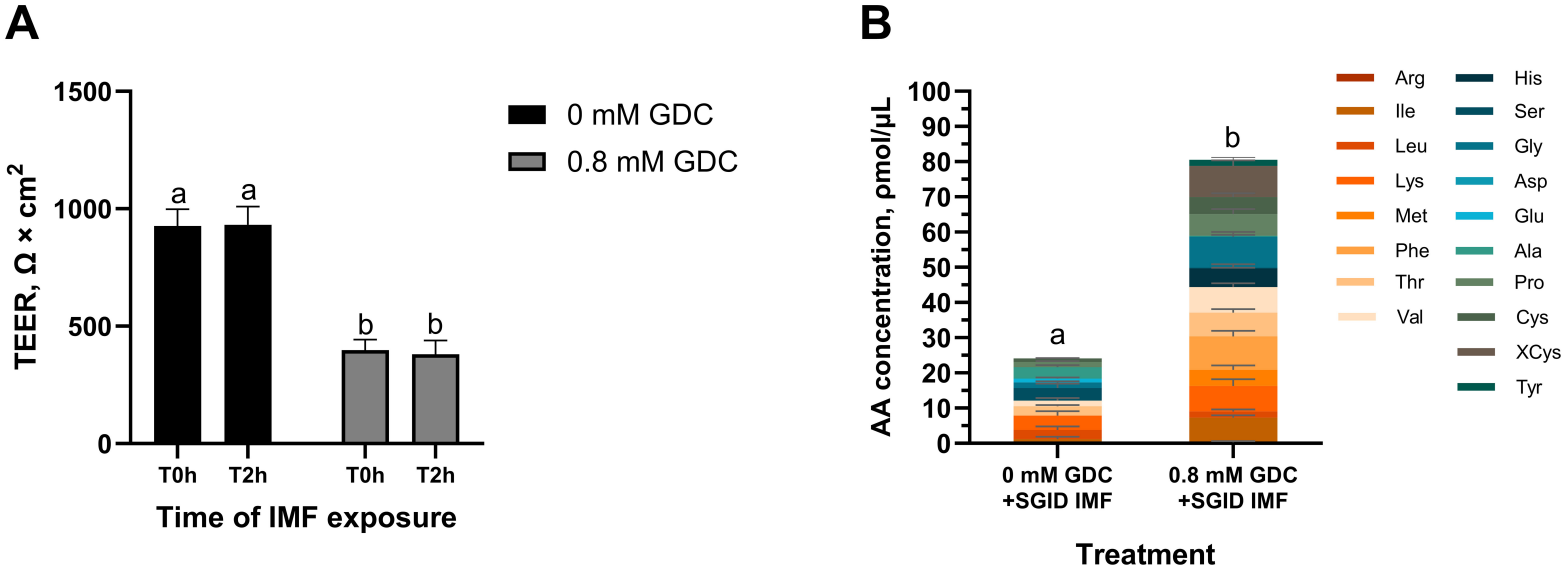
**(A)** TEER values and **(B)** total amino acid content in the basolateral side of Caco-2/HT29-MTX monolayers treated with GDC and digested Infant Milk Formula (IMF). 25-day-old Caco-2/HT29-MTX 90:10 monolayers treated for 2 h with 0.8 mM GDC or DMEM D1145 (0 mM GDC) were washed with HBSS and exposed for 2 h to digested IMF (SGID IMF) diluted 1 in 15 **(A)** TEER values were recorded by Millicell ERS Voltohmmeter after washing (T0h) and after the incubation with the digested IMF (T2h). **(B)** After the 2 h incubation with the IMF digesta, the basolateral samples were subjected to acid hydrolysis and analyzed by UPLC to quantify amino acids. Results represent the average of three biological and two technical replicates ± SEM. Statistical significance (*P* < 0.05) was evaluated using two-way ANOVA with Tukey's multiple comparison **(A)** or Student's *t*-test **(B)**. Different letters indicate significant difference (*P* < 0.05).

Based on permeability, tight junction, mucus data, and timeline to recovery, 0.8 mM GDC was selected as the preferred concentration to create an *in vitro* model of an infant gut barrier. To evaluate whether 0.8 mM GDC-treated monolayers were biocompatible with digested food, IMF was subjected to *in vitro* infant gastrointestinal digestion. This SGID-IMF sample (100.55 ± 3.55 μg protein/cm^2^) was applied to 0.8 mM GDC treated monolayers (Figure 6). Preliminary experiments indicated that this IMF digesta was not toxic to intestinal cells (% of viability = 99.56 ± 2.95, MTS assay, Supplementary Figure 5).

As shown in Figure 6A, no significant differences in TEER values were observed between the T0h and T2h in 0 mM GDC and 0.8 mM GDC monolayers following exposure to SGID IMF (*P* > 0.05), indicating that the monolayer was unaffected by digesta addition. As a control, monolayers were exposed to HBSS alone for 2 h, and no significant change in TEER was observed (TEER T2h vs. T0h = 97.95 ± 6.19% for 0 mM GDC; 106 ± 4.98% for 0.8 mM GDC).

To track nutrient absorption post IMF-digesta treatment, total AA concentration in the basolateral chamber was quantified. Interestingly, total AA concentration in the basolateral chamber was significantly higher in GDC treated monolayers compared to control monolayers (80.55 ± 15.45 ρmol/μL compared to 24.09 ± 8.62 ρmol/μL, *P* < 0.05, Figure 6B).

## 4 Discussion

In our study, we investigated the potential of GDC in converting Caco-2/HT29-MTX monolayers into a model of the infant gut barrier. GDC is a bile salt that has been studied as an epithelial permeation enhancer for oral macromolecule delivery (44, 52). Our main findings are that treatment of Caco-2/HT29-MTX monolayers with 0.8 mM GDC resulted in (a) decreased TEER and increased lactulose paracellular permeability (*P* < 0.05), (b) reduced OCLN at the cell membrane and (c) increased acidic mucins (*P* < 0.05). The monolayer recovered from GDC treatment within 4 h (*P* > 0.05). This timely recovery is essential for an *in vitro* infant gut model designed to test digested foods, as it allows experiments to be performed in buffer rather than culture media. In addition, it simulates food transit times in the upper gut. It is likely that the mechanism of action of GDC involves the activation of G-protein coupled receptors, leading to a reduction in cAMP (*P* < 0.05), with a knock-on effect that includes an increase in extracellular alkaline phosphatase activity (*P* < 0.05) and modulation of intracellular signaling pathways that modify tight junctions. Importantly, our results indicate that neither cytotoxicity (as assessed by MTS and LDH assays, *P* > 0.05), apoptosis (caspase-3 activity, *P* > 0.05), nor inflammation (IL-8, IL-6, and TNF secretion, *P* > 0.05) were involved. GDC treated monolayers were biocompatible with infant formula digesta and resulted in higher concentrations of bioavailable amino acids (*P* < 0.05)

Our findings revealed a significant but transient reduction in TEER and an increase in lactulose Papp (*P* < 0.05) and mRNA levels of the permeability enhancer marker CLDN-2 upon treatment with 0.8 mM GDC. Brayden and Stuettgen (44) reported a similar TEER reduction of 54 ± 13.1% with 0.5 mM GDC in 21/22-day-old Caco-2 cells, (our study = 53.34 ± 25.77% with 0.5 mM GDC). However, Brayden and Stuettgen (44), reported no recovery after 24 h. The inclusion of a mucus layer provides a protective barrier to Caco-2 monolayers (65–67), which possibly supports recovery once GDC is removed.

In agreement with our lactulose Papp data, Song et al. (52) also observed that treating Caco-2 with 2.15 mM GDC increased salmon calcitonin Papp by 10.66-fold and decreased TEER by 4.57-fold, compared to the control (52). Moreover, the Papp/TEER was 2.33 ± 0.56, whilst for the unconjugated form (sodium deoxycholate) the Papp/TEER was 1.31 ± 0.25, indicating the conjugated form is less harmful to the plasma membrane (68). Indeed Brayden and Stuettgen (44) report no significant changes in Caco-2 membrane fluidity after exposure to 0.5 mM GDC using the Laurdan assay (44). In our study, lactulose Papp across 0.8 mM GDC treated monolayers was ~63.35 ± 22.68% higher than control monolayers. Interestingly *in vivo*, lactulose permeability was reported 55.56% higher in the urine of 3-month-old infants (n of samples = 1,715) compared to 15-month infants (n of samples = 1,582). In addition, increased *CLDN2* mRNA has been reported in the proximal small intestine of 2-week-old mice compared to 12-week mice (*P* < 0.05), in line with significantly increased permeability of orally administered 4 kDa, 10 kDa, 70 kDa dextran (*P* < 0.05) (18). However, despite the higher mRNA expression, CLDN-2 protein was localized intracellularly in villi of infant mice indicating, together with short villi, an immature villi architecture (18).

In our study, confocal microscopy revealed that GDC treatment decreased membrane bound OCLN in a dose-dependent manner (*P* < 0.05). Surprisingly, 0.8 mM GDC significantly increased (*P* < 0.05) mRNA expression of tight junction biomarkers OCLN, Claudin-4, JAM-1 (barrier-forming), and Claudin-2 (permeability-enhancing) (19). This suggests that GDC does not downregulate OCLN transcription but instead promotes post-translational modifications, redistribution, and/or internalization of the protein. Similarly, Zeng et al. (69) and Raimondi et al. (57) reported that, in Caco-2 cells, unconjugated deoxycholic acid (0.1–0.3 mM and 50 μM, respectively), induced OCLN post-translational redistribution, potentially through EGFR–mediated internalization (57).

*In vivo* studies have shown that there is a significantly higher level of OCLN leakage in feces at birth compared to 3 and 6-month-old infants (median values: birth = 2.36; 3 months = 2.25; 6 months = 2.17; 12 months = 2.17 ng/g), indicating an increased intestinal permeability correlated to OCLN localization (70). *In vivo* data from animal models show contrasting findings. Holmes et al. (20) report no significant changes in ZO-1, OCLN, and JAM-A mRNA expression in mice during neonatal development (1, 14, 28, and 90 days-old) (20). Whilst, Gleeson et al. (18) reported reduced mRNA levels of ZO-1, OCLN, and JAM-A in 2-week-old infant mice compared to 12-week-old adults.

Our treatment with 0.8 and 1 mM GDC significantly increased acidic mucins (*P* < 0.05), as shown by Alcian blue staining. This stain, binds to sialic acid and sulfate groups of acidic mucins (71). In parallel, the GDC treatment downregulated the expression of mucins MUC-1 (0.5 mM, P < 0.01; 0.8 and 1 mM, P < 0.05) and MUC-2 (P < 0.05), while MUC-5AC remained unchanged (P > 0.05). These findings indicate that there was a shift toward increased acidic mucus that occurred independently from mucin expression.

Several previous studies have shown that bile salts can stimulate mucin secretion. For example, Klinkspoor et al. (72) and Shekels et al. (73) demonstrated that deoxycholic acid (0.0625 to 0.25 mM and 2.5 mM, respectively) and its taurine-conjugated form (taurodeoxycholic acid—2.5 mM) stimulated mucin secretion in a dose-dependent manner in LS174T and HT29 cells (measured by [^3^H]-glucosamine labeling) (72, 73). This effect is believed to be mediated by intracellular calcium and PKC signaling, pathways known to regulate mucin exocytosis in colonic cell lines such as T84 and HT29-18N2 (74–76). Thus, the increased Alcian Blue signal in our study may reflect GDC-induced exocytosis of preformed mucins rather than transcriptional upregulation.

It has also been reported that sodium deoxycholate, glycocholate and taurodeoxycholate (0.2, 0.5, 2 and 20 mM) decrease the viscosity of human-isolated mucus (77). Given GDC's structural similarity to these bile salts, it is likely that GDC similarly reduces mucus viscosity. This, along with increased acidic mucins, may contribute to the infant-like characteristics of our model. *In vivo* data on colonic mucus in newborn humans, pigs and mice show a predominance of acidic mucins over neutral mucins at birth (27). In mice, MUC-2 protein levels and *Muc-3/2/*5 mRNA expression levels progressively increase from 5 to 15 days and at 6 weeks of age (78). Future work would track mucin expression over the GDC recovery period to investigate its alignment to *in vivo* data.

Bile salts primarily interact with intestinal cells through the surface receptor Takeda G protein-coupled Receptor (TGR5) and the nuclear Farnesoid X Receptor (FXR) (55). TGR5 modulates adenylyl cyclase via the G-protein α subunit stimulatory (Gα_s_) or inhibitory (Gα_i_) (79). In our research, we observed a decrease in the intracellular cAMP for the 0.8 and 1 mM GDC-treated monolayers (*P* < 0.05), indicating adenylyl cyclase inhibition and downstream suppression of PKA, pointing to Gα_i_ involvement. Interestingly, TGR5 activation by deoxycholic acid has been linked to ERK1/2 activation, EGFR phosphorylation, and reduced OCLN immunofluorescence in Caco-2, without the involvement of muscarinic receptors, another secondary receptor for bile salts (57, 58). However, conflicting findings from Zeng et al. (69) report a decrease in ERK1/2 phosphorylation in deoxycholic acid-treated Caco-2 cells (69). Although we cannot rule out that GDC modulation could be via alternative β-arrestin or EGFR pathways, the cAMP reduction we observed favors the hypothesis of Gα_i_ involvement (Figure 5C). Furthermore, although nuclear FXR may play a role in this process, its primary effects are though transcriptional changes (55). Therefore, it is unlikely to be the direct cause of the OCLN redistribution induced by GDC as OCLN mRNA levels were unchanged at 0.8 mM GDC. It is also worth noting that while GDC was selected for its low toxicity and reversible effects, other conjugated bile salts such as taurocholate or glycocholate may offer similar advantages. Future studies could explore whether these alternatives yield comparable or complementary effects on epithelial permeability and mucus modulation.

Interestingly, recent insights suggest that intestinal alkaline phosphatase may regulate tight junction protein expression (21). Hamarneh et al. (24) demonstrated that exogenous alkaline phosphatase increases the mRNA levels of ZO-1, ZO-3, and Claudin-3 in Caco-2 cells (24). In our study, GDC at all tested concentrations significantly increased the activity of secreted alkaline phosphatase (*P* < 0.05), suggesting a potential explanation for tight junction mRNA increases observed in our study. This reinforces the idea that alkaline phosphatase may play a role not only in maintaining gut homeostasis, but also in modulating barrier function in early life (25).

While infant formulas aim to replicate breast milk, the latter offers additional bioactive benefits to the infant barrier. For example, milk proteins and their peptides are known to support gut health and nutrient absorption (5). Caco-2/HT29-MTX monolayers treated with 0.8 mM GDC were sufficiently robust to withstand infant formula digesta (food components, digestive enzymes and bile salts). Although GDC was removed after 2 h, the barrier remained moderately permeable for at least 4 h, providing a physiologically relevant window for nutrient absorption kinetics, as intestinal transit in infants typically occurs within this timeframe (orocecal transit time in pre-term infants = 3.1 h) (80). No further TEER decline was observed during digesta exposure, confirming the stability of the model for cellular studies. Most importantly, GDC treated monolayers facilitated higher concentration of AA transported in the basolateral compared to control monolayers (P < 0.05). Although human data are limited, *in vivo* studies in rats and pigs show that the infant barrier allows higher AA transport (81–83). This further demonstrates GDC-treated Caco-2/HT29-MTX suitability as an infant gut barrier model for nutrition research.

One of the model's strengths is its adaptability to compact experimental timelines. Advanced *in vitro* systems must be time-efficient to offer a practical alternative to animal studies. Following the Hubatsch et al. protocol, monolayers are viable and functional from day 21 to 29; thus, a complete experimental sequence—including transient induction of infant-like permeability, followed by repeated exposure to milk components—can be conducted within a focused 8-day window.

While our model provides an infant-like gut barrier, several limitations must be acknowledged. First, compounds can only be tested for a short duration (e.g., 4 h exposure), which may restrict the ability to detect slower or long-term effects on barrier function or improvement. Furthermore, our model currently captures paracellular and amino acids transport only and has not yet been validated for transporter activity or receptor-mediated uptake. It is also crucial to study the potential effects of GDC on brush border enzymes, as well as mucus viscoelastic properties and neutral mucins. Studying neutral mucins will, however, require method development to use Periodic Acid-Schiff with cell lysates. Complementary Alcian Blue and PAS staining post-recovery would help determine if GDC alters acidic and neutral mucin composition through changes in secretion, regulation, or glycosylation. Future studies should also explore whether the conjugated bile salt GDC activates molecular pathways involving β-arrestin, EGFR, and FXR. Additionally, testing the model with breast milk would track its response to breast milk components allowing comparison with *in vivo* data.

## 5 Conclusions

This study proposes 0.8 mM GDC as a suitable treatment for Caco-2/HT29-MTX monolayers to create a reversible, infant-like gut barrier model, which is currently appropriate for infant nutrition research focused on paracellular and AA transport.

## Data Availability

The raw data supporting the conclusions of this article will be made available by the authors, without undue reservation.
