# Supplementary material for: Using sodium glycodeoxycholate to develop a temporary infant-like gut barrier model, in vitro
Source: Front Nutr. 2025 Jun 9;12:1577369. doi: 10.3389/fnut.2025.1577369 (PMC12184380; doi:10.3389/fnut.2025.1577369)

Supplementary Fig. 1: GDC IC_{50} on undifferentiated Caco-2/HT29-MTX cells. Caco-2/ HT29-MTX were seeded at 10^5 cells/well (90:10) and incubated for 24 h in DMEM D1145. Cells were then treated for 2 h with GDC in DMEM D1145. Cell viability was measured as NADPH dehydrogenase activity (MTS assay). The data are expressed as % untreated cells (0 mM GDC). Results are presented as the average of a biological triplicate and a technical duplicate ± SEM.

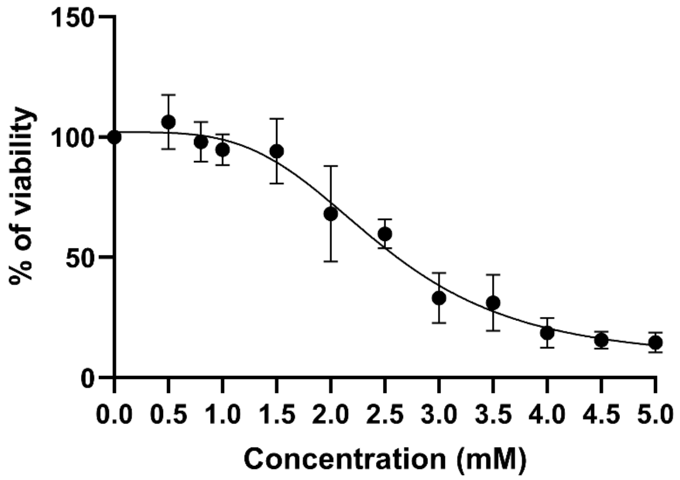

Supplement: Supplementary file 1 [file Image_1.pdf]
