# Supplementary material for: Using sodium glycodeoxycholate to develop a temporary infant-like gut barrier model, in vitro
Source: Front Nutr. 2025 Jun 9;12:1577369. doi: 10.3389/fnut.2025.1577369 (PMC12184380; doi:10.3389/fnut.2025.1577369)

Supplementary Fig. 2: Trans Epithelial Electrical Resistance (TEER) of the (90:10) Caco-2/HT29-MTX monolayers from day 7 to day 25. Cells were seeded at a concentration of 6×10^4 cells/ well in a 12-well Transwell plate. Media was changed every two days and 16 h prior to measurement. TEER values were recorded at day 7, 14, 21, 24 and 25 days by Millicell-ERS Voltohmmeter. Mean TEER value at day 25 was 1302.81 ± 70.32 $\Omega \times cm^2$. Results are presented as the average of a biological triplicate and a technical duplicate ± SEM. Statistical difference between treatments within a time point was assessed by two-way ANOVA with Tukey multiple comparison and is indicated by a different letter ($P < 0.05$).

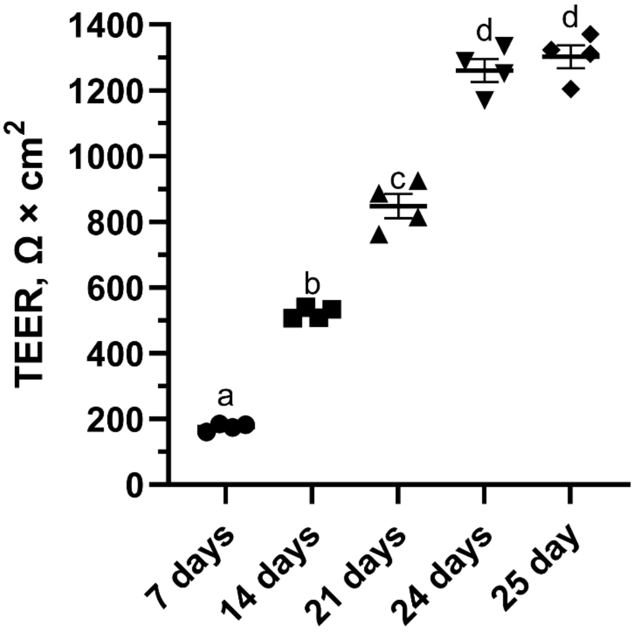

Supplement: Supplementary file 2 [file Image_2.pdf]
