# Supplementary material for: Using sodium glycodeoxycholate to develop a temporary infant-like gut barrier model, in vitro
Source: Front Nutr. 2025 Jun 9;12:1577369. doi: 10.3389/fnut.2025.1577369 (PMC12184380; doi:10.3389/fnut.2025.1577369)

Supplementary Fig. 3: mRNA expression of p21 and p53 as markers to cellular response to stress. Caco-2/HT29-MTX monolayers were seeded at a 90:10 ratio and concentration of 6 × 10^4 cells/ well in a 12-well Transwell plate and cultured for 21 or 25 days. The day prior to the experiment, media was replaced with D1145 and cells were allowed to incubate for approximately 16 hr. Following this, cells were washed with PBS and lysed using RLT buffer. p21 (*CDKN1A*) and p53 (*TP53*) mRNA transcript levels in the cell lysate were quantified using RT-PCR and normalized to the housekeeping gene *RPLP0* mRNA via the $2^{-\Delta\Delta Ct}$ method. Transcript levels are expressed as the fold change relative to the mean value of day 21. .Data represent the mean ± SEM from biological triplicates and technical duplicates. Statistical analysis was conducted using Student's t-test. ns = non-significant difference (P < 0.05).

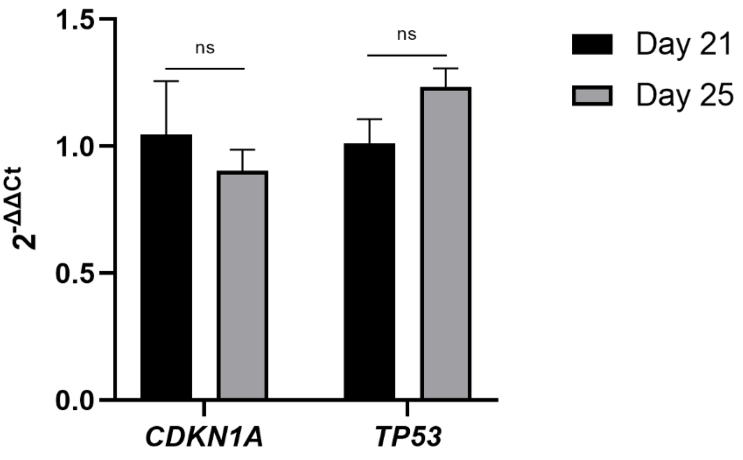

Supplement: Supplementary file 3 [file Image_3.pdf]
