# Supplementary material for: Using sodium glycodeoxycholate to develop a temporary infant-like gut barrier model, in vitro
Source: Front Nutr. 2025 Jun 9;12:1577369. doi: 10.3389/fnut.2025.1577369 (PMC12184380; doi:10.3389/fnut.2025.1577369)

Supplementary Fig. 4: Direct inhibition of Protein Kinase A (PKA) by GDC. GDC solutions (final concentrations = 0.5, 0.8, and 1 mM) were mixed with cAMP (final concentration = 2 μM) in DMEM D1145 + IBMX and incubated with cAMP-Glo™ Assay reagents, including PKA, following the manufacturer instructions. Low cAMP levels signify high PKA activity (increased Relative Light Unit—RLU). Data was generated from a biological triplicate and a technical duplicate ± SEM. Statistical difference in the treatments was assessed by one-way ANOVA with Tukey's multiple comparison and is indicated with different letters ($P < 0.05$).

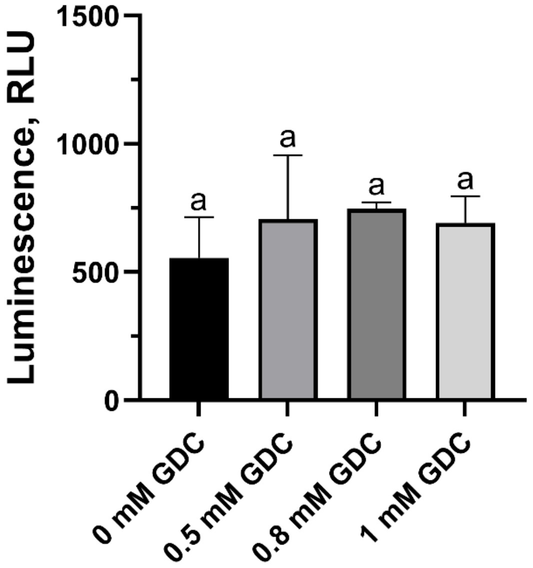

Supplement: Supplementary file 4 [file Image_4.pdf]
