# Supplementary material for: Using sodium glycodeoxycholate to develop a temporary infant-like gut barrier model, in vitro
Source: Front Nutr. 2025 Jun 9;12:1577369. doi: 10.3389/fnut.2025.1577369 (PMC12184380; doi:10.3389/fnut.2025.1577369)

Supplementary Fig. 5: MTS viability assay of undifferentiated Caco-2/HT29-MTX after exposure to digesta. Caco-2/HT29-MTX co-culture (90:10) was seeded at a concentration of 10^5 cells/well in a 96-well plate. After 16 h, cells were treated with IMF (SGID-IMF) or H_2O (SGID H_2O) digesta diluted (1:5, 1:10, 1:15, 1:20) in HBSS for 2 h. Cell viability results are expressed as the mean percentage, with the viability of HBSS-treated cells normalized to 100% (average Abs 490 nm = 0.66 ± 0.017). The mean % of cells in media alone for HBSS-treated cells was 93.79±5.53. SGID IMF = static infant gastrointestinal digestion of Infant milk formula, SGID H_2O = static infant gastrointestinal digestion of water control. Number of biological replicates = 3, technical replicates = 2. Results are mean ± SEM. Asterisk indicates significant difference to HBSS control (P < 0.05).

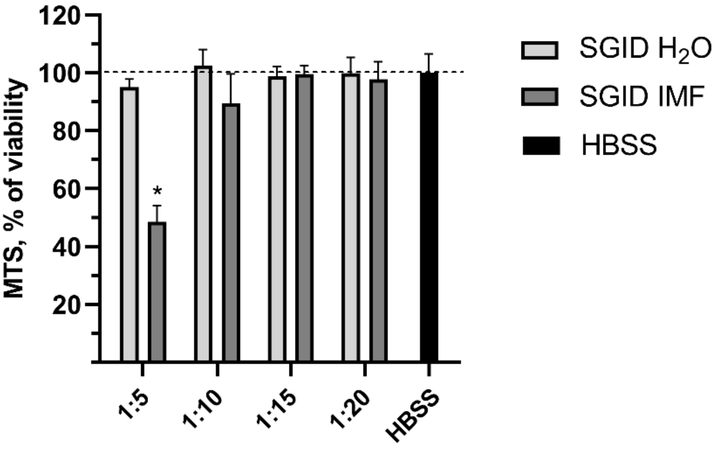

Supplement: Supplementary file 5 [file Image_5.pdf]
