# Supplementary material for: Using sodium glycodeoxycholate to develop a temporary infant-like gut barrier model, in vitro
Source: Front Nutr. 2025 Jun 9;12:1577369. doi: 10.3389/fnut.2025.1577369 (PMC12184380; doi:10.3389/fnut.2025.1577369)
Supplement: Supplementary file 6 [file Table_1.docx]

**Supplementary Table 1: Gene name, GenBank accession number and primers sequences for RT-PCR analysis.**

| **Protein** | **Gene** | **GenBank** | **Foreword Primer** | **Reverse Primer** | **T_a_ (°C)** | **Reference** |
| --- | --- | --- | --- | --- | --- | --- |
| Zonulin-1 | *TJP1* | NM_003257.4 | CATCTCCAGTCCCTTACCTTTC | TCTGCTGGCTTGTTTCTCTAC | 57 | Kondrashina et al. (2021) |
| Occludin | *OCLN* | NM_002538.3 | CTGGCCTACAGGAATACAAGAG | TGATGCTCACAGAGGTTTGG | 57 | Kondrashina et al. (2021) |
| Claudin-2 | *CLDN2* | NM_020384.3 | GCTGAGGATAGACTGACTTTGG | GGGAAGCTTGAGAAGTAGGTTAG | 57 | Kondrashina et al. (2021) |
| Claudin-4 | *CLDN4* | NM_001305.4 | CAGGAATCCAGAGAAACTGGTC | CACCGTGAGTCAGGAGATAAAG | 60 | Kondrashina et al. (2021) |
| Junctional Adhesion Protein-1 | *JAM1* | AF111713.1 | TGTCCTGAATCCCACAACAG | CGGCTATAGGCAAACCAGAT | 57 | Kondrashina et al. (2021) |
| Ribosomal Protein Lateral Stalk Subunit P0 | *RPLP0* | NM_001002.4 | GCAGCATCTACAACCATGAA | GCAGATGGATCAGCCAAGAA | 57 | Kondrashina et al. (2021) |
| Mucin-1 | *MUC1* | NM_002456.6 | CCTACCATCCTATGAGCGAGTAC | GCTGGGTTTGTGTAAGAGAGGC | 60 |  |
| Mucin-2 | *MUC2* | NM_002457.5 | AAGACGGCACCTACCTCG | TTGGAGGAATAAACTGGAGAACC | 60 |  |
| Mucin 5-AC | *MUC5AC* | NM_001304359.2 | ACAGCGGTGACTTCGACACA | CCGATGCCTGCTCCCTGTTA | 60 |  |
| Cyclin-Dependent Kinase Inhibitor 1 (p21) | *CDKN1A* | Not reported | GACACCACTGGAGGGTGACT | CAGGTCCACATGGTCTTCCT | 61 | (Chen et al., 2012) |
| Tumor Protein p53 (p53) | *TP53* | Not reported | GTTCCGAGAGCTGAATGAGG | TGAGTCAGGCCCTTCTGTCT | 61 | (Chen et al., 2012) |
